# Arterial Blood‐Mediated Deep‐Tissue Photoacoustic Oximetry

**DOI:** 10.1002/advs.76366

**Published:** 2026-07-03

**Authors:** Karteekeya Sastry, Junhao Zhu, Joshua Olick‐Gibson, Li Lin, Lei Li, Jigmi Basumatary, Yang Zhang, Lihong V. Wang

**Affiliations:** ^1^ Caltech Optical Imaging Laboratory Andrew and Peggy Cherng Department of Medical Engineering Department of Electrical Engineering California Institute of Technology Pasadena California USA; ^2^ College of Biomedical Engineering and Instrument Science Zhejiang University Hangzhou China; ^3^ Department of Biosciences and Bioengineering Indian Institute of Technology Jammu Jammu India

**Keywords:** arterial blood, biomedical engineering, oxygen saturation, photoacoustic tomography

## Abstract

Photoacoustic tomography is uniquely capable of high‐resolution deep‐tissue blood‐oxygenation (sO_2_) imaging (oximetry) due to its optical absorption contrast. However, wavelength‐dependent optical fluence changes within tissue, i.e., spectral coloring, have impeded the development of photoacoustic oximetry. We present the arterial prior method (APM+; + denotes intravascular fluence correction), which leverages the high arterial sO_2_ to locally calibrate the optical fluence within tissue to circumvent spectral coloring and reliably estimate the sO_2_ near the artery. In phantom experiments with ex vivo animal tissue, APM+ resulted in a median estimation error of 2.9% compared to 9.8% from the traditional linear unmixing method (LUM). In human imaging experiments of the radial artery‐vein pair in eight healthy adult volunteers, the estimated venous sO_2_s from APM+ (median: 72.3%, interquartile range/IQR: 8.9%) were concentrated around the typical 60%–80% range in healthy individuals, whereas those from LUM (median: 75.2%, IQR: 34.4%) varied widely. When imaging the wrists of the eight subjects through ex vivo animal tissue of thicknesses up to 1.5 cm, APM+ provided more consistent estimates than LUM, indicating its robustness with depth.

## Graphical abstract

The arterial prior method (APM+) for photoacoustic oximetry is introduced, which leverages the high arterial blood oxygenation to calibrate the fluence at the artery and estimate the oxygenation of the surrounding tissue, thus circumventing the spectral coloring problem. APM+’s accuracy and consistency are shown in a series of phantom and in vivo human experiments. APM+ is system‐agnostic, depth‐independent, and skin‐tone bias‐free.

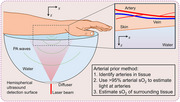

## 1 Introduction

Hemoglobin, a protein in red blood cells, plays a crucial role in delivering oxygen to different parts of the body for cellular respiration, a fundamental process that provides energy for all bodily functions [1, 2]. Thus, the oxygen saturation of hemoglobin in blood (sO_2_), defined as the ratio of oxy‐hemoglobin concentration to the total hemoglobin concentration, is a critical biomarker that offers useful insights into tissue health and metabolic activity. Noninvasive imaging of sO_2_ is vital for several clinical applications, such as monitoring tumor progression [3, 4], studying brain function [5], and, most recently, determining the severity of the infection in coronavirus disease 2019 (COVID‐19) patients [6, 7]. However, existing imaging technologies such as positron emission tomography (PET) and magnetic resonance imaging (MRI) possess key limitations for sO_2_ imaging. PET, for instance, requires radioactive tracers and images tissue hypoxia, rather than providing a quantitative map of sO_2_ in tissue [8, 9]. Similarly, while blood‐oxygen‐level dependent (BOLD) MRI can detect relative changes in sO_2_ [10], the BOLD signal is only sensitive to the deoxy‐hemoglobin concentration [11], and obtaining quantitative estimates of sO_2_ from BOLD MRI involves complex physiological models and remains an area of active research [12].

Optical imaging methods, on the other hand, are naturally suited to noninvasive sO_2_ imaging due to the distinct optical properties of oxy‐ and deoxy‐hemoglobin [13], as evidenced by the bright red and dark red colors of fully oxygenated and deoxygenated blood, respectively. However, high‐resolution optical imaging methods, such as optical coherence tomography [14] (OCT), are typically limited to imaging depths of less than 2 mm due to light scattering [15]. In contrast, while near‐infrared spectroscopy [16] (NIRS) can reach greater depths of up to a few centimeters, it is limited by poor spatial resolution (∼ 1 cm). Beyond imaging, even the most widely used clinical tool, the pulse oximeter [17, 18], which also uses near‐infrared light, has significant drawbacks. It only provides a single estimate of the arterial sO_2_ rather than a complete map of arterial and venous oxygenation in tissue, and it is less accurate for people with darker skin tones [19].

Photoacoustic tomography [20, 21] (PAT) is an emerging optical imaging modality that combines optical excitation with ultrasound detection to enable high‐resolution (< 1 mm) optical imaging well beyond the optical diffusion limit in tissue (∼ 1 mm). In PAT, light from a short laser pulse diffuses into tissue, gets absorbed, and is converted into acoustic waves via thermoelastic expansion in a phenomenon termed the photoacoustic effect [15]. The acoustic waves, which experience low tissue scattering (∼ 1000 times lower than optical scattering), are then recorded using ultrasound transducers surrounding the tissue and used to reconstruct high‐resolution images of the tissue. The contrast in PAT arises from the optical absorption by endogenous chromophores, such as oxy‐ and deoxy‐hemoglobin, making it ideal for noninvasive sO_2_ imaging. However, the reconstructed initial pressure in PAT images is not just proportional to the optical absorption coefficient, but its product with the optical fluence, thus making the photoacoustic signal from an absorber dependent not just on its own absorption properties, but also on the optical properties of the entire tissue [22]. Since the optical properties of the entire tissue change with the optical wavelength, so does the optical fluence distribution within the tissue. This results in a phenomenon termed “spectral coloring” [23, 24], where the photoacoustic spectrum of an absorber differs significantly from its optical absorption spectrum, thus leading to inaccurate sO_2_ estimation beyond superficial depths, where the optical fluence is not known. Overcoming the problem of spectral coloring is key to unlocking PAT's unique ability to perform deep‐tissue, high‐resolution oximetry.

Several approaches have been proposed in the literature to solve the problem of spectral coloring. Many authors have proposed using light propagation models to reconstruct the absorption coefficient within tissue, typically with simplified tissue models [25, 26, 27, 28, 29, 30] or multiple illuminations [31, 32] to ease the ill‐posedness of this problem. However, these methods have not been demonstrated to be effective for in vivo targets, which are often more complex than the assumed tissue models or utilized priors allow. The ill‐posedness can be mitigated by using adjunct imaging data from diffuse optical tomography [33] (DOT) or acousto‐optic tomography [34] (AOT). However, these methods require complicated and expensive hardware modifications and may lack the spatial resolution needed to reliably compensate for the fluence. Tzoumas et al. [35] hypothesized that the fluence spectrum at any point within tissue can be reliably approximated as a linear combination of a few base spectra, which were obtained using simulation. The coefficients of these base spectra were then solved from multi‐wavelength PAT data. Building on this work, Wu et al. [36]. constructed a convex cone of the fluence spectrum by randomly sampling from Monte Carlo simulations of numerous digital phantoms and multiplying it with each possible sO_2_ value. Then, they estimated the sO_2_ at a given location as the one that corresponds to the geometrically closest convex cone to the acquired photoacoustic spectrum at that location. Both these approaches, while impressive, rely on simulations of simple digital phantoms, which may not accurately represent the complexity of in vivo conditions, especially with increasing depth. Moreover, in practice, they require a large number of wavelengths (> 20), which significantly reduces the effective frame rate of sO_2_ imaging and imposes a heavy computational burden. Similarly, several deep learning approaches have been proposed [24, 37, 38, 39], but they are often limited by the need for large labeled datasets, which are typically generated from simulations or simple experimental phantoms. Due to the aforementioned reasons, the de facto method for sO_2_ estimation using PAT is the linear unmixing method [22] (LUM), which ignores the problem of spectral coloring and assumes that the photoacoustic spectrum is identical to the optical absorption spectrum. LUM is preferred primarily due to its simplicity and requirement of only two wavelengths, but its invalid premise of ignoring spectral coloring leads to low accuracy in deeper tissue regions. Therefore, there is a clear need for an sO_2_ estimation method in PAT that is simple to implement, does not require hardware modifications or numerous wavelengths, yet is effective in practice.

To address this pressing need, we propose the arterial prior method (APM). Arteries (except the pulmonary artery) carry highly oxygenated blood from the heart to all parts of the body. In healthy individuals, it is well established that the arterial oxygenation (SaO_2_) is greater than 95% [40]. Therefore, these blood vessels, which are found in all anatomical regions within our body, can be used to calibrate the fluence in the vicinity of the artery. This simple physiological prior lets us circumvent the problem of spectral coloring, thus enabling deep‐tissue photoacoustic oximetry. Since fluence changes occur even within the blood vessel, using the entire artery for calibration leads to biased sO_2_ estimates. To correct this bias, we also introduce a simple intravascular fluence correction approach. APM operates in the image domain and, therefore, is easily implementable across several PAT system configurations. It only requires two wavelengths and does not necessitate any additional hardware modifications to an existing dual‐wavelength PAT system. Although the concept of arterial blood‐based calibration has been introduced previously [41], it has only been tested in numerical simulations and simple experimental phantom demonstrations. Building on this idea, we present APM for deep tissue photoacoustic oximetry and comprehensively test it in phantom and human experiments. We identify the key problem of intravascular fluence variation and introduce a modified APM (denoted by APM+), which corrects the bias APM introduces in sO_2_ estimation. First, we rigorously validate APM and APM+’s accuracy experimentally in phantoms with ex vivo animal tissue to realistically simulate light scattering in tissue. Then, we demonstrate the efficacy of APM+ in vivo in estimating the sO_2_ of the radial vein using the radial artery for calibration in a cohort of 8 healthy adult human subjects. Finally, we demonstrate APM+’s consistency in radial vein sO_2_ estimation through ex vivo animal tissue of thicknesses up to 1.5 cm to simulate tissue depth. Our work firmly establishes APM+ as a simple and effective alternative to the traditional LUM.

## 2 Results

**FIGURE 1:**
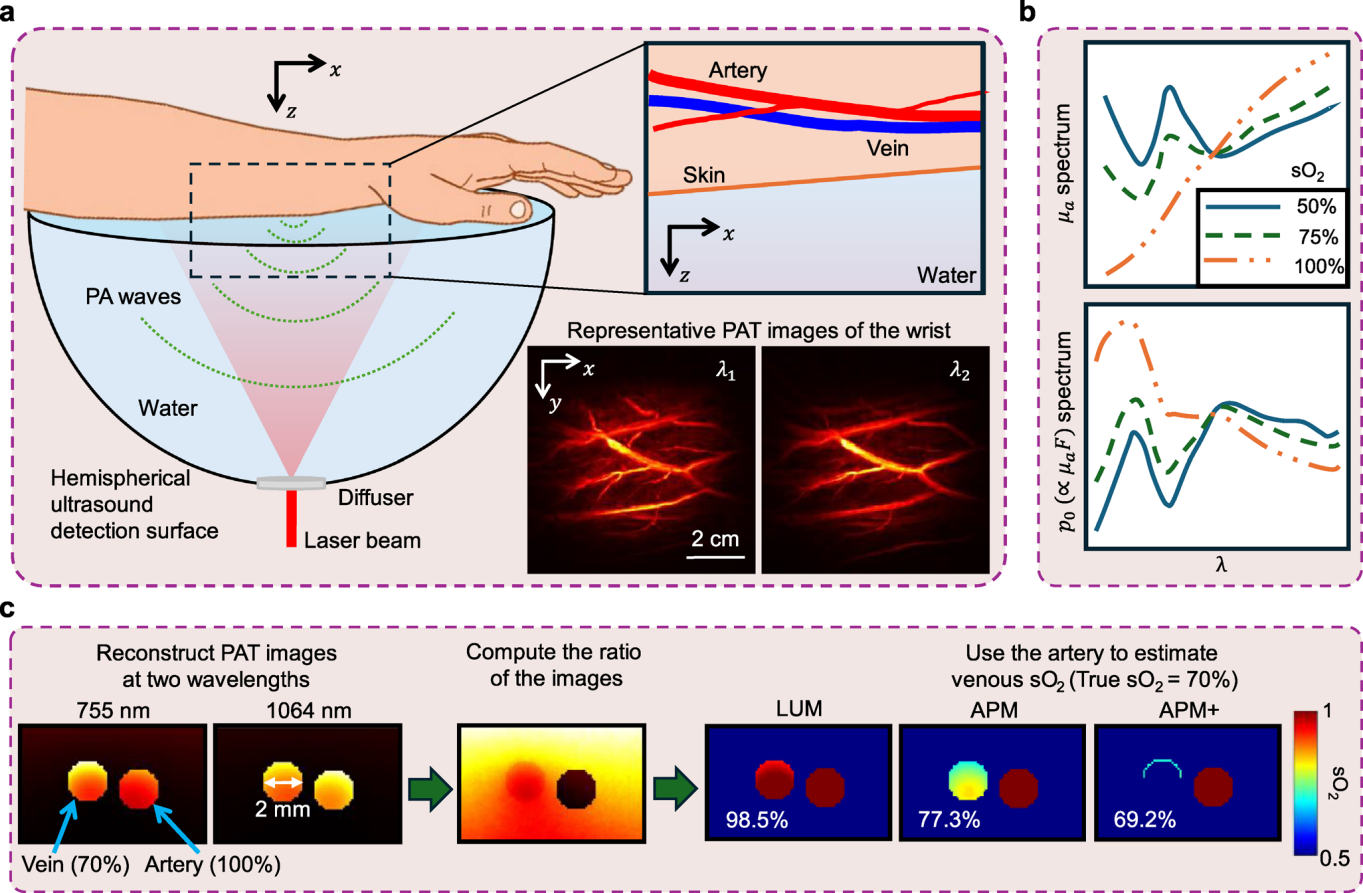
Schematic of arterial blood‐mediated photoacoustic oximetry. (a) (Left) Experimental schematic of PAT of the human wrist in vivo. A ns‐short laser pulse is expanded using a diffuser and delivered to the tissue. The photoacoustic waves generated from the body (shown by green dotted lines) are recorded at a hemispherical ultrasound detection surface surrounding the target. (Top‐right) Cross‐sectional view, showing the skin and an artery‐vein pair in the wrist. The dominant optical absorbers deep within biological tissue in the near‐infrared window are oxy‐ and deoxy hemoglobin contained within the arteries and veins. (Bottom‐right) Representative MAPs of 3D PAT images of the human wrist at two different wavelengths, λ_1_ and λ_2_, respectively. (b) Illustration of spectral coloring. (Top) Representative plot of the optical absorption spectrum of blood with 50%, 75%, and 100% sO_2_, respectively. The variation of the absorption spectrum of blood with sO_2_ enables PAT to estimate blood oxygenation. (Bottom) Representative plot of the initial pressure (*p*_0_) spectrum of blood with 50%, 75%, and 100% oxygenation, respectively. The initial pressure at each wavelength (λ) is a product of the optical absorption coefficient (μ_*a*_) and the optical fluence (*F*) at that wavelength. The *p*_0_ and μ_*a*_ spectra differ significantly in deep tissue due to wavelength‐dependent changes in the optical fluence, i.e., spectral coloring. This makes blood oxygenation estimation from multi‐wavelength PAT images challenging. (c) Workflow of APM for photoacoustic oximetry. We start by reconstructing PAT images of the target at two wavelengths (755 and 1064 nm here). The target is an artery‐vein pair with respective vessel diameters of 2 mm, a center‐to‐center distance of 5 mm between the two vessels, and a depth from the skin surface of 5 mm. The artery and vein have oxygenations of 100% and 70%, respectively. A ratio of the images at the two wavelengths is taken to cancel out any wavelength‐independent factors. Finally, the venous sO_2_ is estimated using APM, assuming the arterial sO_2_ to be known. Additionally, a correction is performed for the intra‐vascular fluence variation in APM+, and the estimated venous sO_2_ using LUM, APM, and APM+ are 98.5%, 77.3%, and 69.2%, respectively. The spatial sO_2_ maps from LUM, APM, and APM+, respectively, are shown on the right. They illustrate a clear trend in the predicted sO_2_ with depth in the case of APM, which is remedied with the correction in APM+. LUM, on the other hand, significantly overestimates the venous sO_2_ everywhere within the vessel.

The concept of APM+ for arterial blood‐mediated photoacoustic oximetry is illustrated in Figure 1. Figure 1a shows an experimental schematic of PAT of the human wrist. A laser pulse is expanded using a diffuser and delivered to the tissue to be within the American National Standards Institute (ANSI) safety limit [42]. The laser light results in the emission of photoacoustic waves, which are recorded at a hemispherical ultrasound detection surface surrounding the tissue. Since the most dominant optical absorbers within biological tissue in the near‐infrared (NIR) window (650–1350 nm) are oxy‐ and deoxy‐hemoglobin [13], the primary structures of interest in photoacoustic images are arteries and veins, as illustrated in the top‐right of Figure 1a. Representative maximum amplitude projections (MAPs) of the three‐dimensional (3D) PAT images of the wrist of a human subject at two wavelengths, λ_1_ and λ_2_, respectively, are shown in the bottom‐right of Figure 1a. Due to the distinct optical absorption spectra of oxy‐ and deoxy‐hemoglobin, blood with different levels of oxygen saturation (sO_2_) has distinct optical absorption spectra, as illustrated in the representative plot in the top of Figure 1b. Thus, optical absorption measurements of blood can be used to estimate the sO_2_ of the blood. However, PAT images represent the initial pressure distribution in tissue, *p*_0_, which is proportional to the product of the optical absorption coefficient (μ_*a*_) and the optical fluence (*F*) at each wavelength. Since the optical fluence distribution at each wavelength depends on the optical properties of the tissue everywhere, the *p*_0_ spectrum is markedly different from the μ_*a*_ spectrum, as illustrated in the representative plot in the bottom of Figure 1b. This phenomenon, termed spectral coloring, makes photoacoustic oximetry in deep tissue challenging.

In APM, we propose to calibrate the fluence within tissue by leveraging the known physiological insight that in healthy individuals, arterial blood is highly oxygenated (sO_2_> 95%). The key steps in the workflow of APM are illustrated in Figure 1c using numerical simulations. First, the PAT images of a two‐dimensional (2D) digital phantom are reconstructed at two wavelengths, respectively (755 and 1064 nm here). The digital phantom comprises an artery‐vein pair at a depth of 5 mm from the skin surface, with each vessel having a diameter of 2 mm and a distance between the centers of two vessels of 5 mm. The true sO_2_s of the artery and vein are 100% and 70%, respectively. Next, a ratio of the images at the two wavelengths is taken to cancel out any wavelength‐independent factors, such as the Grüneisen parameter, the total hemoglobin concentration, the spatial sensitivity of the transducer array, etc. Finally, the venous sO_2_ is estimated using the traditional LUM as well as APM. The mean estimated sO_2_s obtained using LUM and APM are 98.5% and 77.3%, respectively, indicating the higher accuracy of APM. The residual bias in the APM estimate is primarily attributed to the fluence variation within the artery. A correction is performed to account for this effect by first only considering the top edge of the vessel, which is least impacted by light propagation through the vessel, and then performing a coarse correction using a simulated fluence variation map (see Methods). APM with intravascular fluence correction, denoted by APM+, results in a mean sO_2_ of 69.2%, which is much closer to the ground truth (70%). The spatial maps of the estimated venous sO_2_ using LUM, APM, and APM+ are shown in Figure 1c. A clear trend is observed with depth in the spatial map corresponding to APM, which is remedied using the intravascular fluence correction in APM+.

**FIGURE 2:**
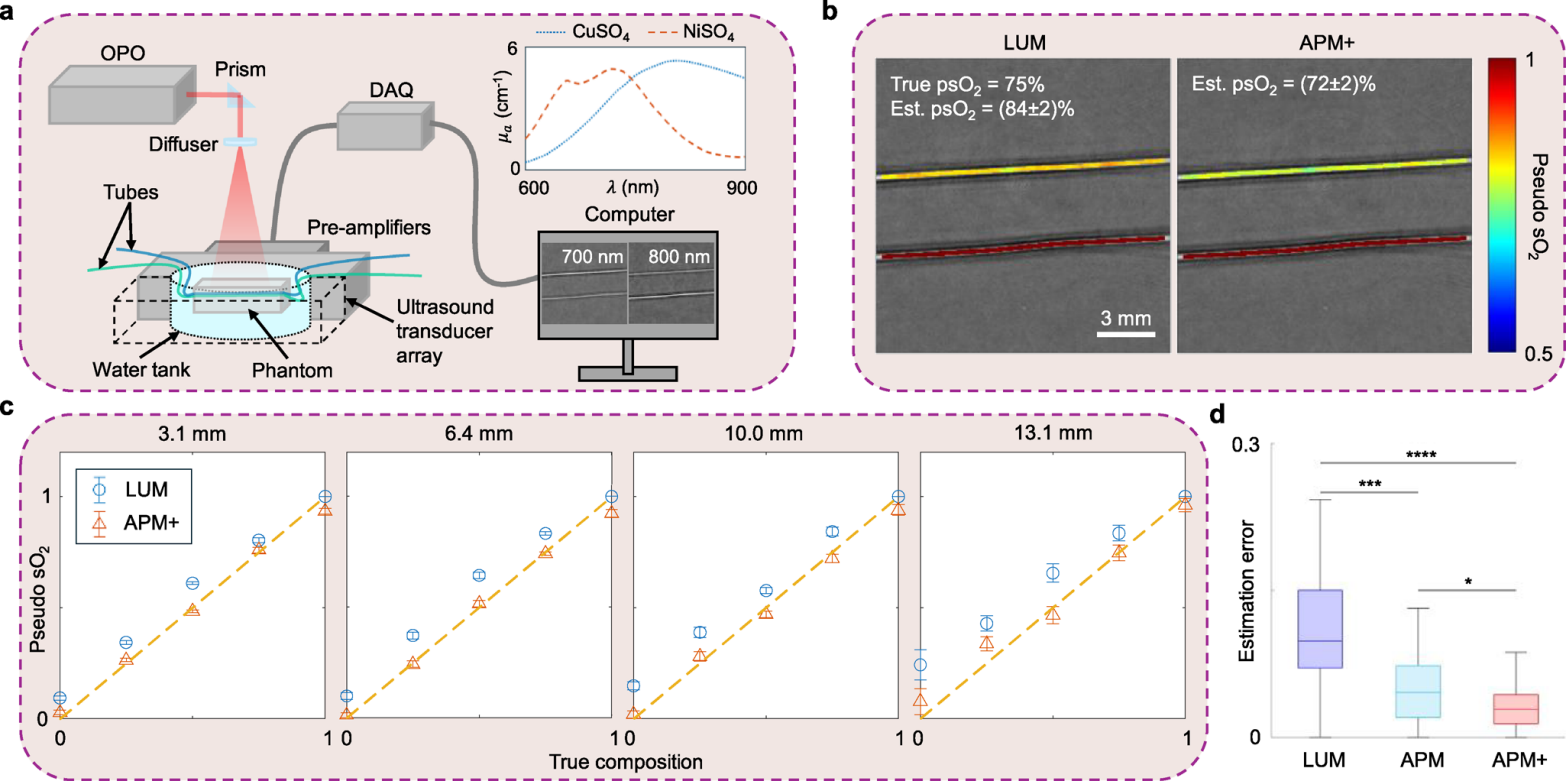
Phantom validation of APM+. (a) Experimental schematic of the phantom validation. An agarose phantom with two parallel embedded microrenathane tubes (5 mm separation) is imaged. One tube, which mimics an artery, contains 100% 0.2 m CuSO_4_ solution, whereas the other tube, which mimics a vein, contains mixtures of 0.2 m CuSO_4_ and 1.2 m NiSO_4_ solutions in different ratios to achieve various pseudo‐sO_2_ (psO_2_) levels. The phantom is illuminated by light pulses from an optical parametric oscillator (OPO) at wavelengths of 700 and 800 nm, respectively, which are expanded using an engineered diffuser. The generated photoacoustic waves are received by a ring‐shaped ultrasound transducer array, acquired by pre‐amplification and data acquisition modules (DAQ), and finally streamed to a computer, where the PAT images are reconstructed. Ex vivo porcine tissue of varying thicknesses, ranging from 3 to 13 mm, is placed on top of the phantom to simulate the spectral coloring encountered in human tissue in vivo. (Top‐right) Plot of the optical absorption spectra of 0.2 m CuSO_4_ and 1.2 m NiSO_4_. (b) Estimated LUM and APM+ psO_2_ maps overlaid on the corresponding PAT image of the phantom at 800 nm with a true venous psO_2_ of 75% and a depth of 10.0 mm. The estimated venous psO_2_s using LUM and APM+ are 84 ± 2% and 72 ± 2%, respectively, indicating a much lower error for APM+. (c) Plots of estimated venous psO_2_s using LUM and APM+ for different true psO_2_s and ex vivo tissue thicknesses. The APM+ estimates are much closer to the ground truth (yellow dashed line) than the LUM ones. (d) Plot of the estimation error distributions for LUM, APM, and APM+, respectively. Paired‐sample t‐tests revealed statistically significant differences between each pair of the three error distributions (sample size: 20, ^*^: *p* < 0.05, ^***^: *p* < 0.001, ^****^: *p* < 0.0001).

We validated the accuracy of APM using phantom experiments with ex vivo animal tissue to simulate spectral coloring in deep tissue. The experimental schematic for the phantom validation is shown in Figure 2a. Light from an optical parametric oscillator (OPO) is diffused onto two parallel micro‐renathane tubes (inner diameter: 0.6 mm, outer diameter: 1 mm, Braintree Scientific) with a separation of 5 mm, embedded in 3% agarose (A9414, low gelling temperature, MilliporeSigma). The emitted photoacoustic waves from the phantom are received by a 512‐element ring‐shaped elevationally‐focused ultrasound array with a center frequency of 5 MHz, amplified by a pre‐amplification module, digitized by a data acquisition module (DAQ), and streamed to a computer, where the PAT images are reconstructed. The two tubes are filled with solutions of 0.2 m CuSO_4_ and 1.2 m NiSO_4_, which mimic oxy‐ and deoxy‐hemoglobin, respectively [43]. Their optical absorption spectra are shown in the top‐right of Figure 2a. One of the tubes, meant to mimic an artery, is filled with 100% CuSO_4_ solution, whereas the other tube contains a mixture of the two solutions with a pseudo‐sO_2_ (psO_2_) between 0% and 100%, where psO_2_ is defined as the percentage by volume of the 0.2 m CuSO_4_ solution in the mixture. Ex vivo porcine tissues of thicknesses ranging from 3 to 13 mm are placed on top of the agarose phantom to simulate spectral coloring found in biological tissue, and PAT images of the phantom are acquired at 700 and 800 nm, respectively. Finally, the psO_2_ of the mixture in the “venous” tube is estimated using LUM, APM, and APM+, respectively.

Representative psO_2_ maps obtained using LUM and APM+, respectively, for a true psO_2_ of 75% and an ex vivo tissue thickness of 10.0 mm are shown in Figure 2b. While LUM overestimated the (mean ± standard error) psO_2_ as (84 ± 2) %, APM+ results in a more accurate estimate of (72 ± 2) %. The mean estimated psO_2_s for different true psO_2_s and ex vivo tissue thicknesses are shown in Figure 2c. The APM+ estimates are much closer to ground truth (yellow dashed line) than the LUM ones. We also plot the distributions of the estimation errors from LUM and APM+ in Figure 2d (sample size = 20). The median APM+ error was 2.9% (upper quartile/Q3: 4.4%), which was significantly lower than the LUM median error of 9.8% (Q3: 15%). We also plot the errors for APM (without intravascular fluence correction; median: 4.6%, Q3: 7.3%), which still performs significantly better than LUM, but is worse than APM+. We performed paired‐sample t‐tests, which revealed statistically significant differences between errors from LUM and APM+ (*p* < 0.0001), LUM and APM (*p* < 0.001), and APM and APM+ (*p* < 0.05), respectively. The descriptive statistics for the three error distributions in Figure 2d are summarized in Table S1. This phantom demonstration establishes the superior accuracy of APM+ compared to LUM for photoacoustic oximetry, even at depths beyond 1 cm.

Having established the accuracy of APM+, we proceed to demonstrate it in humans in vivo. We imaged the radial artery and the two accompanying radial veins in the wrists of eight healthy adult human volunteers using a 3D PAT system [44] at wavelengths of 755 and 1064 nm, respectively, and predicted the sO_2_ of the radial veins using LUM and APM+, respectively. Since the imaged volunteers are healthy adults, we expect the venous sO_2_ to mostly be between 60% and 80% [22, 40, 45]. Further, since the accompanying radial veins drain blood from the same anatomical region, we expect the sO_2_ of the two radial veins to be similar.

**FIGURE 3:**
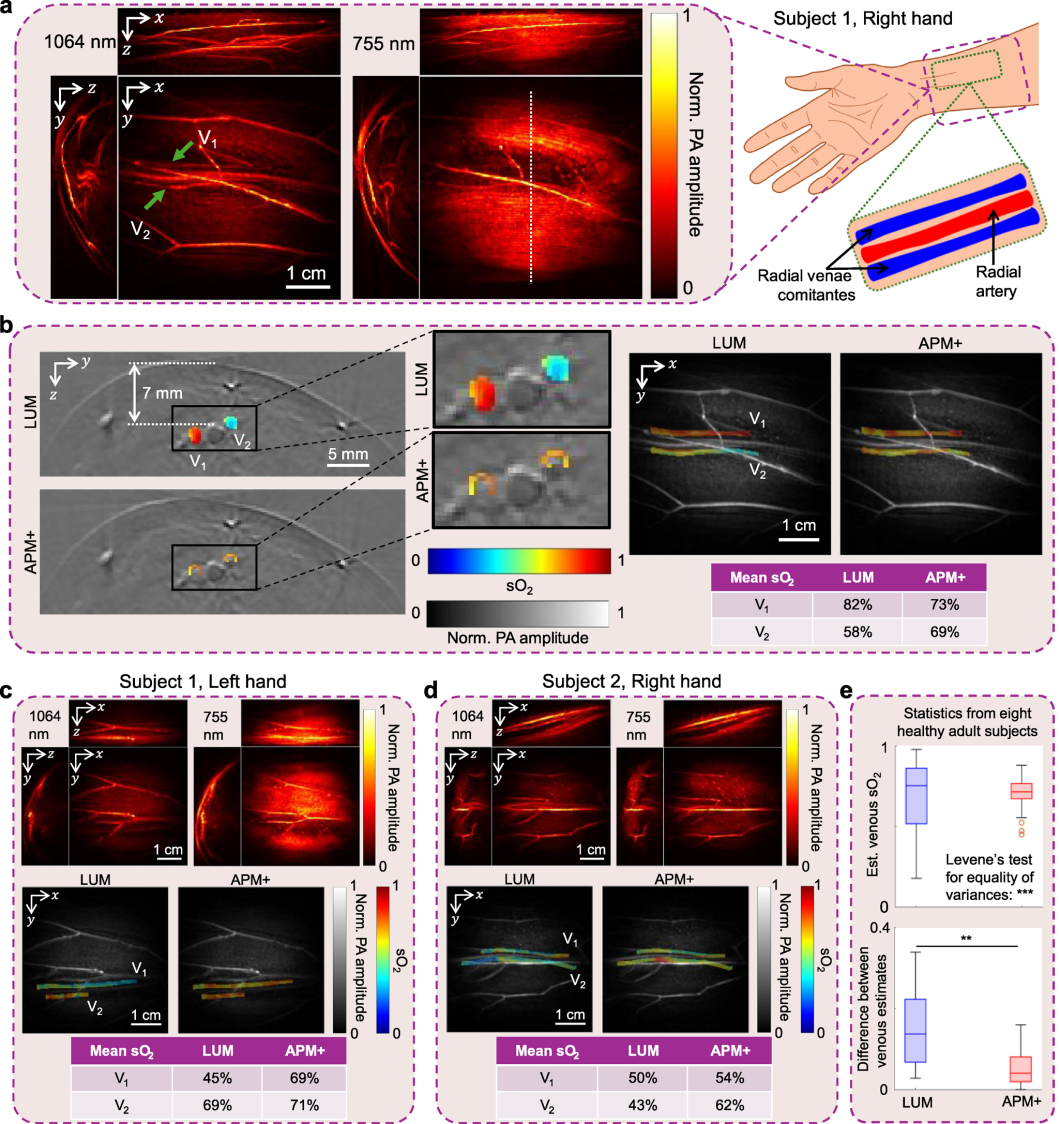
In vivo estimation of radial vein oxygenation in adult human subjects. (a) Orthogonal MAPs of PAT images of the right wrist of a healthy adult human subject at 755 and 1064 nm, respectively. The vessels of interest within this field‐of‐view are the radial artery and the accompanying radial veins on either side of the artery (radial venae comitantes), as illustrated in the bottom‐right. The radial venae comitantes are identified in the 1064 nm photoacoustic (PA) image using green arrows. (b) (Left) Cross‐sectional PAT image, corresponding to the slice indicated by the white dotted line in (a), overlaid with the estimated venous sO_2_ maps of the radial veins using LUM and APM+, respectively. The depth of the radial artery from the skin surface in this cross‐section is approximately 7 mm. While the LUM estimates for the two accompanying radial veins are significantly different, the APM+ estimates are noticeably closer to each other, owing to the arterial calibration. (Center) Magnified views of the respective LUM and APM+ estimated sO_2_ maps for clarity. (Right) Mean projection of the 3D estimated venous sO_2_ maps from LUM and APM+ overlaid on the *x*‐*y* MAP of the 1064 nm PAT image. The mean estimated sO_2_s for vessels V_1_ and V_2_ using LUM are 82% and 58%, respectively, whereas those from APM+ are 73% and 69%, respectively. (c) Results from the left wrist of the same subject. The mean estimated sO_2_s for vessels V_1_ and V_2_ using LUM are 45% and 69%, respectively, whereas those from APM+ are 69% and 71%, respectively. (d) Results from the right wrist of a second subject. The mean estimated sO_2_s for vessels V_1_ and V_2_ using LUM are 50% and 43%, respectively, whereas those from APM+ are 54% and 62%, respectively. (e) Summary statistics for all the subjects (8 subjects, 15 wrist images, 30 vessels). (Top) Plot of the estimated sO_2_s of the radial veins of 8 healthy adult human subjects obtained using LUM and APM+, respectively. The APM+ estimates are consistently within the 60%–80% expected range for healthy subjects, whereas the LUM estimates span a much wider range. Outliers are samples that are more than 1.5 times the interquartile range away from the first or third quartiles. A Levene's test revealed that the variances of the distributions were statistically different (sample size: 30, ^***^: *p* < 0.001; Cohen's *d*  = 0.925 for absolute deviations from the median; *d* ≥ 0.8 indicates a large effect size). (Bottom) Plot comparing the distributions of the sO_2_ difference between the two paired radial veins, as estimated by LUM and APM+, respectively, for all subjects. This difference is significantly lower on average (sample size: 20, ^**^: *p* < 0.01, paired‐samples *t*‐test; Cohen's *d*  = 0.845) for APM+ compared to LUM, as expected for the two radial veins draining blood from the same anatomical region.

Representative orthogonal MAPs of 3D PAT images of the (right) wrist of a healthy adult human volunteer at 755 and 1064 nm, respectively, are shown in Figure 3a. The radial artery and venae comitantes (accompanying veins) run nearly parallel in the wrist, as illustrated in the bottom‐right of Figure 3a. The radial venae comitantes in the 1064 nm PAT image are identified with green arrows as V_1_ and V_2_. We estimate the sO_2_s of the radial venae comitantes in the images presented in Figure 3a using LUM and APM+ (assuming an arterial sO_2_ of 97%), respectively, and report the results in Figure 3b. The estimated sO_2_ maps for the radial veins using LUM and APM+, respectively, overlaid on a cross‐section of the PAT image (indicated by the dotted white line in Figure 3a), are shown on the left in Figure 3b. The radial artery in this cross‐section is at a depth of ∼ 7 mm from the skin surface. We see that the LUM estimates for the two radial veins are notably different from each other, likely due to shadowing from a superficial vessel directly over V_2_, whereas the APM+ estimates are nearly identical. Magnified views of the sO_2_ maps of the radial veins are shown in the center of Figure 3b for clarity. The mean estimated sO_2_s of V_1_ and V_2_ (shown in the bottom‐right of Figure 3b) using LUM are 82% and 58%, respectively, whereas those using APM+ are 73% and 69%, respectively, further demonstrating the consistency of APM+. The spatial sO_2_ maps of the two veins, obtained using LUM and APM+, respectively, overlaid on the *x*‐*y* MAP of the 1064 nm PAT image, are also shown in the top‐right of Figure 3b.

The sO_2_ estimation results corresponding to the subject's left wrist and the right wrist of a second healthy adult human subject are shown in Figure 3c,d, respectively. They show the reconstructed dual‐wavelength PAT images for each case in the top row, the respective estimated sO_2_ maps in the center row, and the estimated mean sO_2_s for the two radial veins using LUM and APM+ in the bottom row. For the left wrist of subject 1, the LUM estimates for V_1_ and V_2_ are 45% and 69%, respectively, whereas those using APM+ are 69% and 71%, respectively. Similarly, for the right wrist of subject 2, the LUM estimates for V_1_ and V_2_ are 50% and 43%, respectively, whereas those using APM+ are 54% and 62%, respectively. Finally, the mean estimated sO_2_s from eight healthy subjects (8 subjects, 15 wrist images, 30 vessels) are summarized in Figure 3e. The dual‐wavelength images corresponding to the left hand of one of the subjects were discarded due to poor image quality. The top plot in Figure 3e shows the estimated venous sO_2_s across all subjects (sample size: 30), whereas the bottom plot shows the difference between the estimated sO_2_s of the radial venae comitantes in each dual‐wavelength image (sample size: 15) obtained using LUM and APM+, respectively. We see that while the APM+ estimates (median: 72.3%, Q1: 68.6%, Q3: 77.5%) lie quite tightly within the expected 60%–80% range in healthy individuals, the LUM estimates (median: 75.2%, Q1: 51.7%, Q3: 86.1%) vary much more widely. A Levene's test confirmed that the variances of the two distributions were significantly different (*p* < 0.001; Cohen's *d*  = 0.925 for absolute deviations from the mean). Similarly, the difference between the sO_2_s of the venae comitantes is significantly lower for the APM+ estimates (median: 4.0%, Q3: 8.0%) compared to the LUM (median: 13.8%, Q3: 22.3%) ones, as confirmed by a paired‐sample t‐test (*p* < 0.01; Cohen's *d*  = 0.845). The descriptive statistics for the box‐plots in Figure 3e are summarized in Table S2. A comparison of the venous sO_2_ estimates from APM and APM+ (without and with intravascular fluence correction, respectively) across all subjects is provided in Figure S1. Lastly, we also verified that performing LUM with fluence compensation (using literature‐derived tissue properties [13]: blood volume fraction = 5%, sO_2_ = 65%, water volume fraction = 10%) did not significantly decrease the variation in the LUM estimates.

**FIGURE 4:**
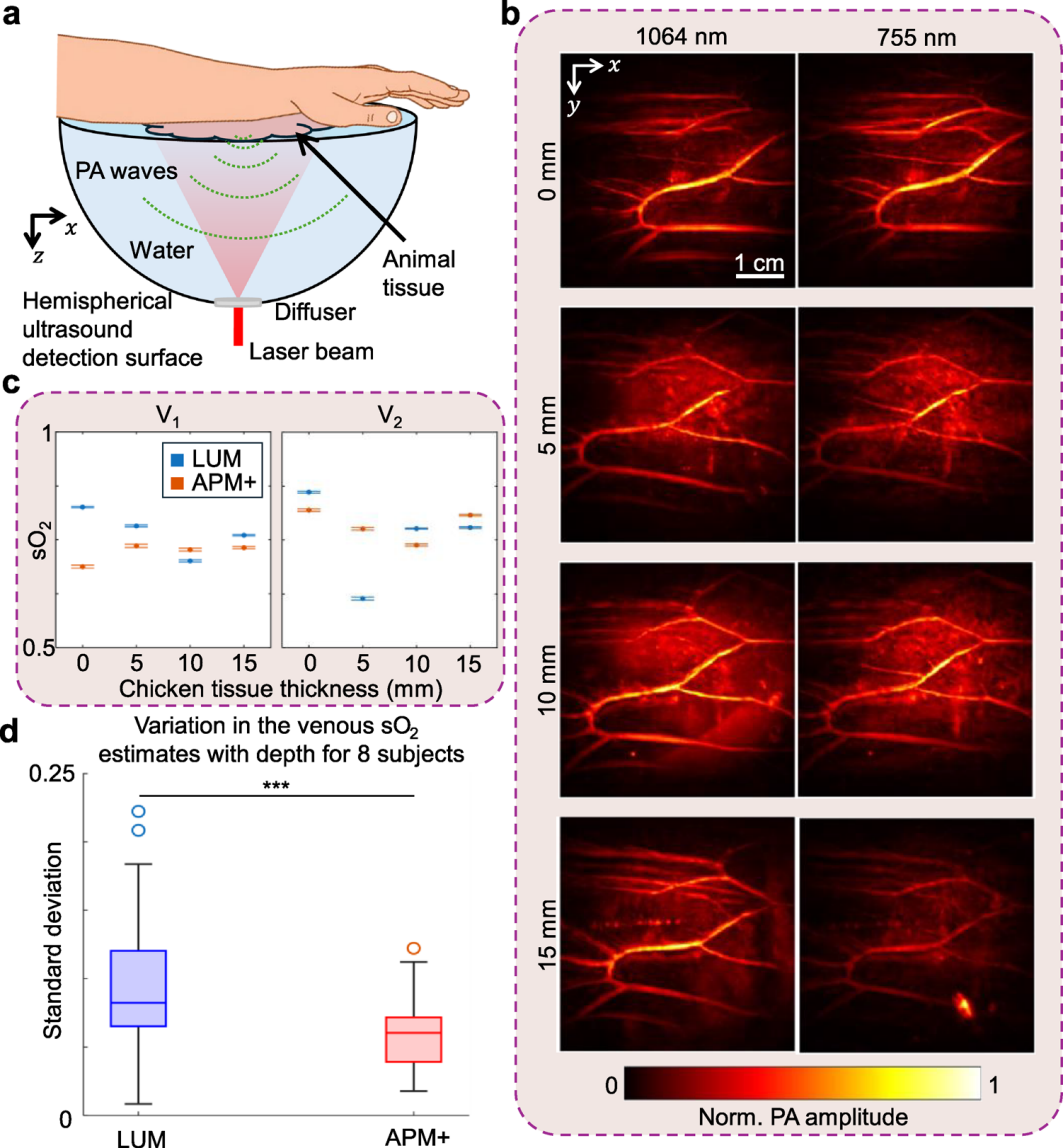
In vivo consistency demonstration of APM+ using ex vivo animal tissue. (a) Experimental schematic for in vivo consistency demonstration. The wrist is imaged first without any ex vivo tissue and then with 5, 10, and 15 mm of ex vivo chicken tissue, respectively. The sO_2_s of the radial veins are estimated for each depth using LUM and APM+, respectively, and the variation with depth for the two methods is compared. (b) MAPs of representative PAT images of the wrist of a healthy human subject at 755 and 1064 nm, and with ex vivo tissue thickness of 0, 5, 10, and 15 mm, respectively. (c) Plot of the estimated mean sO_2_s of the accompanying radial veins, V_1_ and V_2_, corresponding to the images shown in (b). The APM+ estimates show significantly less variation with tissue thickness compared to LUM. The total variation in each of the APM+ estimates was 3% (V_1_) and 8% (V_2_), whereas that from LUM was 10% (V_1_) and 20% (V_2_), respectively. (d) Plot of the standard deviation with depth for 8 healthy adult human subjects (15 wrist images, 30 vessels) of the LUM and APM+ venous sO_2_ estimates. Outliers are samples that are more than 1.5 times the interquartile range away from the first or third quartiles. The variation of the APM+ estimates is significantly lower on average than the LUM ones, as confirmed by a paired‐sample *t*‐test (sample size: 30, ^***^: *p* < 0.001; Cohen's *d*  = 0.804).

To further test the consistency of APM+, we imaged the wrists of the 8 healthy adult volunteers through ex vivo chicken tissue of thicknesses of 5, 10, and 15 mm, respectively. The different tissue samples induce distinct optical fluence distributions for the same target within the tissue and simulate tissue depth. We then estimate the sO_2_s of the radial veins using LUM and APM+, respectively, for each ex vivo tissue thickness. The variation in these estimates with depth informs us about the consistency and robustness of each method. A schematic of the experimental setup showing the expanded laser pulse, the imaging target along with the ex vivo tissue, the photoacoustic wave emission, and the hemispherical ultrasound detection surface is shown in Figure 4a. MAPs of representative 3D PAT images at 1064 and 755 nm, respectively, for each ex vivo tissue thickness are shown in Figure 4b. The estimated sO_2_s for the two radial veins, V_1_ and V_2_, in the images shown in Figure 4b are plotted in Figure 4c, and they show that the variation in the LUM estimates with depth is larger than that of the APM+ ones. For each vessel, we compute the standard deviation with depth of the sO_2_ estimates for LUM and APM+, respectively, for the 8 healthy adult human subjects (sample size: 30) and show them in Figure 4d. As mentioned previously, the images corresponding to the left hand of one of the subjects were discarded due to poor image quality. The median and Q3 of the LUM estimates are 8.2% and 12%, respectively, whereas those of the APM+ estimates are 6.2% and 7.4%, respectively. A paired‐sample t‐test revealed a significant difference between the means of the two distributions (*p* < 0.001; Cohen's *d*  = 0.804). The descriptive statistics corresponding to the box‐plots in Figure 4d are given in Table S3. A comparison of the standard deviations of the venous sO_2_ estimates from LUM, APM, and APM+, respectively, across all subjects is provided in Figure S2.

## 3 Discussion

We presented APM for deep‐tissue photoacoustic oximetry. By leveraging known information about the high oxygenation of arterial blood, APM circumvents the spectral coloring problem that limits the traditional LUM. Further, we identified intravascular fluence variation as a key source of error in arterial fluence calibration and introduced APM+, which included a correction step to improve APM's accuracy. We demonstrated the superior accuracy of APM+ over LUM in a controlled ex vivo phantom experiment and its enhanced consistency and robustness relative to LUM across a series of in vivo human experiments. Phantom experiments with ex vivo animal tissue revealed that APM+ resulted in a much lower median pseudo‐sO_2_ estimation error of 2.9% compared to the 9.8% obtained from the LUM method. We also showed that incorporating the intravascular fluence correction made a significant difference to the estimation accuracy. To establish the clinical potential of APM+, we applied it to dual‐wavelength 3D PAT images of the wrists of eight healthy adult human volunteers and estimated the oxygenation of the radial veins. While the mean predicted sO_2_s of the two radial veins across eight subjects using APM+ (median: 72.3%, interquartile range/IQR: 8.9%) were concentrated around the expected 60%–80% range in healthy individuals [22, 40, 45], those from LUM (median: 75.2%, IQR: 34.4%) spanned a much wider range. Furthermore, the differences between the estimated sO_2_s of the two radial venae comitantes from APM+ (median: 4.0%) are significantly lower than those from LUM (median: 13.8%). Finally, we imaged the wrists of 8 healthy adult human volunteers through 0, 5, 10, and 15 mm of ex vivo animal tissue, respectively, and showed that the mean estimated sO_2_s from APM+ exhibit much lower variation than those from LUM, thus elucidating the consistency and robustness of APM+ with depth. These demonstrations firmly establish the superior accuracy and consistency of APM+ over the traditional LUM.

APM+ (and APM) holds several advantages over existing approaches in the literature that address spectral coloring. First, unlike several existing approaches that require numerous wavelengths [35, 36], thus increasing acquisition times, APM+ only requires two wavelengths, which results in a higher frame rate for sO_2_ imaging. Second, since APM+ is an image‐domain method that only requires reconstructed dual‐wavelength PAT images of the target, it is easily adaptable across PAT systems of various configurations without needing any hardware modifications. For example, it could be readily applied to several existing studies that perform oxygenation measurements of different artery‐vein pairs in the body [46, 47, 48, 49, 50] to address important clinical problems. Third, since APM+ relies on arterial calibration, its accuracy does not depend on the imaging depth (provided an artery is present at a similar depth) or the subject's skin tone, as shown in the simulation study in Note S1. Finally, APM+ is widely applicable since arteries perfuse all parts of our body, and most major arteries in our body have accompanying veins (e.g., the brachial artery‐vein pair in the arm, the carotid artery‐internal jugular vein in the neck), whose sO_2_ estimates can potentially also be combined with blood flow measurements [51, 52] in the future to obtain the metabolic rate of oxygen consumption (MRO_2_) of different organs.

APM+ (and APM) also has some limitations. First, it requires the presence of an artery in the imaging field of view. While this requirement is readily satisfied in most anatomical regions, the method is only effective in the vicinity of an artery. Further, its accuracy can be affected if, for example, a superficial vessel causes shadowing around an artery, thus creating an irregular fluence pattern around it. Second, for APM+, it is necessary to have a reliable method for identifying arteries in PAT images. In the current work, we used the anatomical knowledge of the wrist to identify the radial artery‐vein pair. It is also possible to leverage the pulsatility of arterial blood flow to identify arteries, as demonstrated previously [53]. Third, APM+ performs intravascular fluence correction to improve the arterial fluence estimation accuracy by assuming literature‐derived tissue properties [13]. While we have verified that the method is robust to deviations from these assumed parameters (see Methods), developing an intravascular fluence correction approach that does not require this assumption would further enhance the method's generality. Finally, APM+ requires prior knowledge of the arterial sO_2_. While this is not a concern in healthy individuals, whose SaO_2_ is known to be within the narrow range of 95%–100%, in individuals who are critically ill, the SaO_2_ can drop significantly. In these cases, in the absence of a more accurate method for obtaining the SaO_2_, a pulse oximeter may be used to obtain a noninvasive estimate of the SaO_2_. An analysis of the spatial validity of our method and the impact of SaO_2_ error on its estimation accuracy is provided in Note S2.

With several PAT platforms worldwide having recently obtained regulatory approvals [21, 54, 55, 56] for the clinical application of PAT in humans, the technology is on the cusp of widespread clinical adoption. Thus, the timely development of APM+ for noninvasive, deep‐tissue photoacoustic oximetry will potentially lead to improved diagnosis and monitoring across a wide spectrum of diseases. In oncology, where aggressive tumor growth is often linked to tissue hypoxia [4], monitoring the tissue oxygenation might provide critical information for the early detection of tumors. For diabetic patients, poor circulation can lead to slow‐healing wounds and ulcers, particularly in the extremities, which can even result in amputations in extreme cases [44, 57]. Monitoring tissue oxygenation in these patients might help track wound recovery and assess the efficacy of treatment options. Transcutaneous oxygen pressure monitoring [58] (TcPO_2_) is one of the standard diagnostic tools used to diagnose peripheral vascular disease (PVD). However, TcPO_2_ measurements are superficial and are affected by temperature and edema [59]. Instead, monitoring the sO_2_ and blood flow [52, 60] in deep tissue using PAT could offer a comprehensive and versatile tool for detecting PVD. Future studies that demonstrate our method's efficacy in assessing and monitoring these conditions will be key to its clinical translation.

The performance of APM+ can potentially be improved further. First, the pulsatility of arteries [53] can be leveraged to reliably identify all the arteries in the image. With the complete arterial network identified, fluence calibration can be performed over a much larger part of the tissue, and more sophisticated interpolation and inpainting techniques [61] can be utilized to predict the sO_2_ accurately over the entire imaged region. With arteries identified at multiple depths in the tissue, the optical properties of the background tissue may also be estimated to increase the depth validity of the method. Further, layered ex vivo phantoms that accurately model different layers of tissue, such as skin, fat, muscle, and other inhomogeneities, such as vascular inclusions to emulate superficial vessels, may also help better characterize the performance of APM+ in controlled settings. Second, the illumination wavelengths can be optimally chosen to improve the robustness of the method to SaO_2_ errors and fluence ratio heterogeneity, as described in Note S3. Lastly, while the current work has comprehensively demonstrated APM+’s accuracy in phantoms and consistency in healthy volunteers, a study of the in vivo accuracy of the method, potentially using invasive blood‐gas measurements of venous blood as the gold standard, is envisioned as the next step towards clinical translation. Nevertheless, we show in Note S4 that in the current study, APM+ achieves a higher in vivo accuracy than LUM, provided the estimation error of each method is statistically independent of the true sO_2_. Although this assumption is not strictly true due to the boundedness of the true sO_2_, based on the phantom results in Figure 2, we show that this yields a conservative lower bound for the difference between the accuracies of the two methods.

In conclusion, we have introduced APM for deep‐tissue photoacoustic blood oximetry, which leverages the high oxygenation of arterial blood to estimate the blood oxygenation in the vicinity of the artery. We also introduced APM+ by modifying APM, which incorporates a correction scheme for intravascular fluence changes and improves APM's accuracy. APM+ is system‐agnostic, robust to spectral coloring in deep tissue, requires no additional hardware modifications to existing dual‐wavelength PAT systems, and therefore has the potential to be widely adopted in practice. We expect that APM+ will supplant the traditional LUM method for photoacoustic oximetry, wherever arterial calibration is feasible, and will open several exciting clinical avenues for PAT, thus accelerating its progress towards mainstream clinical adoption.

## 4 Methods

### 4.1 Data Acquisition

The phantom data in Figure 2 were acquired using the 2D PAT system described here [62], with a ring‐shaped 512‐element ultrasound transducer array (Imasonics Inc.; diameter: 10 cm; center frequency: 5 MHz; one‐way 6 dB bandwidth: ∼90%). Light of wavelengths 700 and 800 nm, respectively, from an OPO (SpitLight EVO III, InnoLas Laser; pulse repetition rate: 100 Hz; 5–8 ns pulse width, pulse energy: 23.5 mJ at 700 nm, 18.4 mJ at 800 nm) was expanded using an engineered diffuser (EDC‐10‐A‐1r, RPC Photonics Inc.) and delivered to the target. The received photoacoustic waves were amplified by a pre‐amplification module and digitized using a fully parallel data acquisition system (Sonix, Ultrasonix Medical ULC). The data were then streamed to a computer through a universal serial bus (USB) 3.0 for image reconstruction and analysis. Photoacoustic signals from 25 laser pulses (0.25 s) were averaged to ensure a sufficient signal‐to‐noise ratio (SNR) for the phantom images.

The in vivo data in Figures 3 and 4 were acquired using the 3D PAT system described here [44], with a synthetic hemispherical ultrasound detection surface obtained by rotating arc‐shaped arrays (Imasonic Inc.; bowl diameter: 26 cm; center frequency: 2.25 MHz; one‐way 6 dB bandwidth: ∼100%). 1064 nm light from an Nd:YAG laser (LPY7875, Litron; pulse repetition rate: 10 Hz; pulse energy: ∼1.6 J; pulse width: 4–7 ns) and 755 nm light from an Alexandrite laser (Alex‐Q, Beamtech Optronics Co., Ltd.; pulse repetition rate: 10 Hz; pulse energy: ∼0.4 J; pulse width: 70 ns), respectively, were expanded using an engineered diffuser (EDC‐40, RPC Photonics Inc.) to be within the ANSI safety limit [42] and delivered to the tissue in an interleaved manner. The emitted photoacoustic waves were received by the ultrasound array, amplified by a pre‐amplification module, digitized by a data acquisition system (Photosound Inc.; maximum sampling rate: 40 MHz; dynamic range: 12 bits), and finally streamed to a computer using USB 3.0. The lasers, DAQ, and scanning motor were synchronized using National Instruments LabVIEW (64‐bit). A single acquisition of dual‐wavelength PAT data was completed within 5 s to ensure sufficient measurement density while minimizing motion artifacts.

### 4.2 Linear Unmixing

Under conditions of thermal and stress confinement, the initial pressure (*p*_0_; Pa) that is set up in tissue upon irradiation is proportional to the product of the local optical absorption coefficient (μ_*a*_; m^−1^) and the optical fluence (F; Jm^−2^), i.e., *p*_0_∝μ_*a*_ *F*. The most prominent absorbers in biological tissue in the NIR window (650–1350 nm) are oxy‐ and deoxy‐hemoglobin. The optical absorption coefficient of tissue in these wavelengths can therefore be expressed as:

(1) $$\mu_{a}\;\left(\lambda \right)=c_{\mathrm{Hb}}\;\times \varepsilon_{\mathrm{Hb}}\left(\lambda \right)+c_{\mathrm{Hb}O_{2}}\times \varepsilon_{\mathrm{Hb}O_{2}}\left(\lambda \right)$$

where *c*_Hb_ and $c_{\mathrm{Hb}O_{2}}$ are the concentrations (M) of deoxy‐ and oxy‐hemoglobin, respectively, and ε_Hb_(λ) and $\varepsilon_{\mathrm{Hb}O_{2}}\left(\lambda \right)$ are the molar extinction coefficients (cm^−1^M^−1^) of deoxy‐ and oxy‐hemoglobin, respectively. Thus, assuming that the fluence distribution is known, *c*_Hb_ and $c_{\mathrm{Hb}O_{2}}$ can be readily deduced from PAT images at two different wavelengths, as shown below:

(2) $$\left[\begin{array}{c} c_{\mathrm{Hb}}\\ c_{\mathrm{Hb}O_{2}} \end{array}\right]={\left[\begin{array}{cc} \varepsilon_{\mathrm{Hb}}\left(\lambda_{1}\right) & \varepsilon_{\mathrm{Hb}O_{2}}\left(\lambda_{1}\right)\\ \varepsilon_{\mathrm{Hb}}\left(\lambda_{2}\right) & \varepsilon_{\mathrm{Hb}O_{2}}\left(\lambda_{2}\right) \end{array}\right]}^{-1}\;\left[\begin{array}{c} p_{0}\left(\lambda_{1}\right)/F\left(\lambda_{1}\right)\\ p_{0}\left(\lambda_{2}\right)/F\left(\lambda_{2}\right) \end{array}\right]$$

The sO_2_ can then be estimated as the ratio of the oxy‐hemoglobin concentration to the total hemoglobin concentration, i.e.,

(3) $$sO_{2}=\frac{c_{\mathrm{Hb}}}{c_{\mathrm{Hb}}+c_{\mathrm{Hb}O_{2}}}\;$$

Alternatively, if the fluence at the two wavelengths is assumed to be unknown but identical, the fluence term can be ignored in Equation (2) since it would cancel out in the ratio in Equation (3). This approach is termed the linear unmixing method (LUM), and it is widely used in the literature due to its simplicity.

### 4.3 Arterial Prior

A key limitation of LUM is its assumption of a known or wavelength‐independent fluence distribution in tissue, which is invalid due to the unknown and wavelength‐dependent tissue optical properties. While normalizing for the surface fluence at each wavelength is a common practice, it becomes inaccurate beyond the optical diffusion limit (∼1 mm), since the surface fluence does not accurately reflect the fluence in deep tissue. To overcome this fundamental barrier of spectral coloring, we propose the arterial prior method (APM), which uses the known properties of arterial blood to estimate the sO_2_ in the vicinity of the artery. To derive APM, we start with the relation between the reconstructed initial pressure, the fluence, and the sO_2_.

(4) $$\begin{array}{cc} & {\hat{p}}_{0}\;\left(\vec{r},\lambda \right)\\ & \;=S\left(\vec{r}\right)F\left(\vec{r},\lambda \right)\left(\varepsilon_{\mathrm{Hb}O_{2}}\left(\lambda \right)sO_{2}\left(\vec{r}\right)+\varepsilon_{\mathrm{Hb}}\left(\lambda \right)\left(1-sO_{2}\left(\vec{r}\right)\right)\right) \end{array}$$

where ${\hat{p}}_{0}\left(\vec{r},\lambda \right)$ is the reconstructed PAT image as a function of the position, $\vec{r}$, and illumination wavelength, λ, $F(\vec{r},\lambda )$ is the optical fluence distribution, and $S(\vec{r})$ is a term that captures the combined effect of several wavelength‐independent multiplicative factors, such as the total hemoglobin concentration, the Grüneisen parameter, the spatially varying point spread function of the PAT system, etc. Next, consider the ratio of the PAT images at two wavelengths, λ_1_ and λ_2_:

(5) $$\begin{array}{cc} \frac{{\hat{p}}_{0}\left(\vec{r},\lambda_{1}\right)}{{\hat{p}}_{0}\left(\vec{r},\lambda_{2}\right)}= & \frac{F\left(\vec{r},\lambda_{1}\right)}{F\left(\vec{r},\lambda_{2}\right)}\;\times \;\frac{\mu_{a}\left(\vec{r},\lambda_{1}\right)}{\mu_{a}\left(\vec{r},\lambda_{2}\right)}=\frac{F\left(\vec{r},\lambda_{1}\right)}{F\left(\vec{r},\lambda_{2}\right)}\;\\ & \;\times \frac{\varepsilon_{\mathrm{Hb}O_{2}}\left(\lambda_{1}\right)sO_{2}\left(\vec{r}\right)+\varepsilon_{\mathrm{Hb}}\left(\lambda_{1}\right)\left(1-sO_{2}\left(\vec{r}\right)\right)}{\varepsilon_{\mathrm{Hb}O_{2}}\left(\lambda_{2}\right)sO_{2}\left(\vec{r}\right)+\varepsilon_{\mathrm{Hb}}\left(\lambda_{2}\right)\left(1-sO_{2}\left(\vec{r}\right)\right)} \end{array}$$

Assuming that the sO_2_ of an artery is known, the ratio of the fluences at the two wavelengths in an arterial location, ${\vec{r}}_{\mathrm{art}}$, can be computed as:

(6) $$\frac{F\left({\vec{r}}_{\mathrm{art}},\lambda_{1}\right)}{F\left({\vec{r}}_{\mathrm{art}},\lambda_{2}\right)}=\frac{{\hat{p}}_{0}\left({\vec{r}}_{\mathrm{art}},\lambda_{1}\right)}{{\hat{p}}_{0}\left({\vec{r}}_{\mathrm{art}},\lambda_{2}\right)}\;\times \frac{\mu_{a}\left({\vec{r}}_{\mathrm{art}},\lambda_{2}\right)}{\mu_{a}\left({\vec{r}}_{\mathrm{art}},\lambda_{1}\right)}$$

Then, at $\vec{r}$ in the vicinity of the artery, we have:

(7) $${\hat{\mu }}_{R}\left(\vec{r}\right)≔\frac{{\hat{p}}_{0}\left(\vec{r},\lambda_{1}\right)}{{\hat{p}}_{0}\left(\vec{r},\lambda_{2}\right)}\times \frac{F\left({\vec{r}}_{\mathrm{art}},\lambda_{2}\right)}{F\left({\vec{r}}_{\mathrm{art}},\lambda_{1}\right)}$$

where ${\hat{\mu }}_{R}\left(\vec{r}\right)$ is an estimate of the ratio $\mu_{R}\;\left(\vec{r}\right)=\frac{\mu_{a}\left(\vec{r},\lambda_{1}\right)}{\mu_{a}\left(\vec{r},\lambda_{2}\right)}$. Finally, the sO_2_ in the vicinity of the artery can be estimated as:

(8) $$s{\hat{O}}_{2}\;\left(\vec{r}\right)={\left(1-\frac{{\hat{\mu }}_{R}\left(\vec{r}\right)\varepsilon_{\mathrm{Hb}O_{2}}\left(\lambda_{2}\right)-\varepsilon_{\mathrm{Hb}O_{2}}\left(\lambda_{1}\right)}{{\hat{\mu }}_{R}\left(\vec{r}\right)\varepsilon_{\mathrm{Hb}}\left(\lambda_{2}\right)-\varepsilon_{\mathrm{Hb}}\left(\lambda_{1}\right)}\right)}^{-1}\;$$

### 4.4 Intravascular Fluence Correction

The optical fluence at each wavelength exhibits variations within the vessel lumen, which, if not accounted for, result in inaccuracies in the sO_2_ estimation using APM. For the 3D PAT images in Figures 3 and 4, we performed a two‐step correction. First, we only used the top edge of each vessel since these voxels would be least influenced by light propagation through the vessel. Then, to correct for residual fluence variation across the vessel edge, a re‐weighting of the artery was performed at each wavelength. The re‐weighting uses Monte Carlo simulated fluence variation maps [63] of the artery at each wavelength, normalized to the fluence at the top‐most voxel of the artery. Although these properties (except the SaO_2_) are not known a priori, we found in practice that performing the second step with canonical tissue optical properties [13] (Background tissue: blood volume fraction = 5%, sO_2_ = 65%, water volume fraction = 10%; Artery: sO_2_ = 97%, c_HbT_ = 15 g/dL) resulted in better estimation performance, as shown in Figure S1, which provides a comparison of the in vivo results in Figure 3e without (APM), with only the first step (denoted by APM*), and with both steps (APM+) of the intravascular fluence correction. Nevertheless, since this step introduces a dependence on the assigned tissue parameters, we have verified that a ± 20% change (relative to the values mentioned above) in the tissue blood volume fraction and sO_2_ results in a relatively small change in the median and IQR of the in vivo APM+ estimates in Figure 3e of less than ± 1% and ± 0.5%, respectively. For the 2D phantom demonstration in Figure 2, where we image the central slice of the vessels, a simple 1D Beer's law‐based correction was carried out only for the tube with 100% CuSO_4_.

### 4.5 Image Reconstruction and Data Analysis

The PAT image reconstruction was performed using the universal backprojection (UBP) method [64]. Since the UBP method results in bipolar reconstructed images primarily due to the band‐limitedness of the ultrasound transducers, we perform UBP on the analytical signal and take the absolute value of the resulting complex‐valued image. For each in vivo photoacoustic image (isotropic voxel size: 0.25 mm), the radial artery and venae comitantes were manually segmented. Spatial smoothing with a 1.5‐mm diameter spherical kernel was performed before applying LUM and APM+, respectively, to estimate the sO_2_. The fluence ratio at each venous location was estimated as the average arterial fluence ratio around the nearest arterial location. Morphological operations were performed on the segmented vessel masks to extract the vessel edges for intravascular fluence correction. The optical fluence within the artery was obtained from Monte Carlo simulations performed using the MCXLAB toolbox [63]. The surface fluence at each wavelength was obtained for LUM by measuring the fluence without any water in the hemispherical bowl and accounting for water propagation losses in the water. An arterial sO_2_ of 97% was assumed for the in vivo demonstrations in Figures 3 and 4. The impact of errors in the arterial sO_2_ on APM's accuracy is discussed in Note S2. The image reconstruction and data analysis were carried out in MATLAB R2025a running on a Windows 10 computer with an Inter Core i7‐8700 at 3.2 GHz, 3 × 16 GB DDR4 RAM at 2666 MHz, and an NVIDIA GeForce GTX 1050 Ti GPU.

### 4.6 Imaging Protocols

All human imaging experiments were performed with the relevant guidelines and regulations approved by the Institutional Review Board of the California Institute of Technology (Caltech). They were performed in a dedicated imaging room. Written informed consents were obtained from all the participants, who were made aware that their data would be deidentified in any publication, in accordance with the study protocol (IR22‐1187).

## Author Contributions

L.V.W. and K.S. designed the study. K.S., J.Z., J.O.G., L.L., L.L., J.B., and Y.Z. performed the experiments. K.S. and J.Z. recruited the human subjects. K.S. analyzed the data and wrote the manuscript with input from all the authors. L.V.W. supervised the study and revised the manuscript.

## Conflicts of Interest

L.V.W. has a financial interest in Microphotoacoustics Inc., CalPACT LLC, and Union Photoacoustic Technologies Ltd., which, however, did not support this work.

## Supporting information




**Supporting File**: advs76366‐sup‐0001‐SuppMat.docx.

## Data Availability

The data that support the findings of this study are provided within the paper and its supplementary material.
