# Supplementary material for: Arterial Blood‐Mediated Deep‐Tissue Photoacoustic Oximetry
Source: Adv Sci (Weinh). 2026 Jul 3:e76366. Online ahead of print. doi: 10.1002/advs.76366 (PMC13334587; doi:10.1002/advs.76366)
Supplement: Supplementary file 1 — Supporting File: advs76366‐sup‐0001‐SuppMat.docx. [file ADVS-9999-e76366-s001.docx]

**Supplementary material for:**

**Arterial blood-mediated deep-tissue photoacoustic oximetry**

Karteekeya Sastry^1^, Junhao Zhu^1^, Joshua Olick-Gibson^1^, Li Lin^1,2^, Lei Li^1^, Jigmi Basumatary^1^, Yang Zhang^1^, Lihong V. Wang^1,^*

^1^ Caltech Optical Imaging Laboratory, Andrew and Peggy Cherng Department of Medical Engineering, Department of Electrical Engineering, California Institute of Technology, 1200 East California Boulevard, Pasadena, CA 91125, USA

^2^ Present address: College of Biomedical Engineering and Instrument Science, Zhejiang University,
Hangzhou, 310007, China

*Corresponding author: Lihong V. Wang (LVW@caltech.edu)

**List of supplementary materials**

Table S1| Descriptive statistics corresponding to Figure 2d

Table S2| Descriptive statistics corresponding to Figure 3e

Table S3| Descriptive statistics corresponding to Figure 4d

Figure S1| Comparison of APM+, APM*, and APM in-vivo

Figure S2| Comparison of in-vivo consistency of LUM, APM+, and APM.

Note S1| Impact of skin tone on APM+.

Figure S3| Demonstration of the skin tone bias-free nature of APM+.

Note S2| Error analysis of APM.

Figure S4| Analysis of the sO_2_ estimation error due to fluence ratio variation.

Note S3| Choosing the best wavelengths for APM.

Figure S5| Impact of wavelength choice on APM.

Note S4| Analysis of the in-vivo accuracies of APM+ and LUM.

**Supplementary Materials**

**Table S1| Descriptive statistics corresponding to Figure 2d**

(Error in the estimated psO_2_)

| **Sample size = 20** | **LUM** | **APM** | **APM+** |
| --- | --- | --- | --- |
| **Median** | 9.8% | 4.6% | 2.9% |
| **Lower quartile (Q1)** | 7.1% | 2% | 1.4% |
| **Upper quartile (Q3)** | 15% | 7.3% | 4.4% |
| **Interquartile range (IQR = Q3 - Q1)** | 7.9% | 5.3% | 3 % |
| **Maximum** | 24.3% | 13.2% | 8.7% |

**Table S2| Descriptive statistics corresponding to Figure 3e**

(Top plot: Estimated venous sO_2_)

| **Sample size = 30** | **LUM** | **APM+** |
| --- | --- | --- |
| **Median** | 75.2% | 72.3% |
| **Lower quartile (Q1)** | 51.7% | 68.6% |
| **Upper quartile (Q3)** | 86.1% | 77.5% |
| **Interquartile range (IQR = Q3 - Q1)** | 34.4% | 8.9% |
| **Maximum** | 97.8% | 88.4% |
| **Minimum** | 17.8% | 56.7% |
| **Range (max – min)** | 80% | 31.7% |

(Bottom plot: Difference between venous estimates of accompanying radial veins)

| **Sample size = 15** | **LUM** | **APM+** |
| --- | --- | --- |
| **Median** | 13.8% | 4% |
| **Lower quartile (Q1)** | 6.8% | 2% |
| **Upper quartile (Q3)** | 22.3% | 8% |
| **Interquartile range (IQR = Q3 - Q1)** | 15.5% | 6% |
| **Maximum** | 33.9% | 16% |
| **Minimum** | 2.8% | 0% |
| **Range (max – min)** | 31.1% | 16% |

**Table S3| Descriptive statistics corresponding to Figure 4d**

(Variation in the venous sO_2_ estimates with depth)

| **Sample size = 30** | **LUM** | **APM+** |
| --- | --- | --- |
| **Median** | 8.2% | 6.2% |
| **Lower quartile (Q1)** | 6.5% | 4.1% |
| **Upper quartile (Q3)** | 12% | 7.4% |
| **Interquartile range (IQR = Q3 - Q1)** | 5.5% | 3.3% |
| **Maximum** | 18.4% | 11.5% |
| **Minimum** | 0.8% | 1.8% |
| **Range (max – min)** | 17.6% | 9.7% |


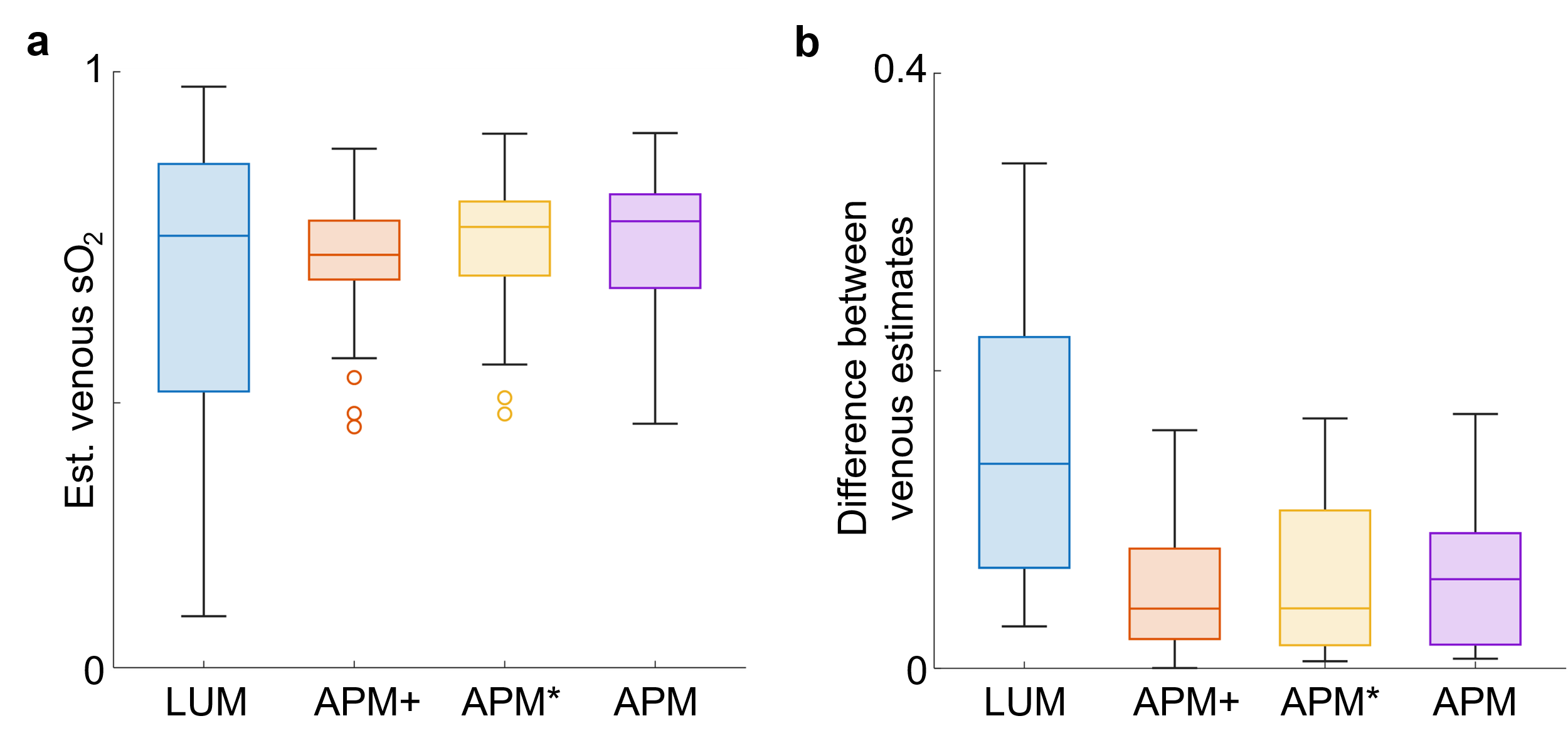


| **Figure S1\| Comparison of APM+, APM*, and APM in-vivo.** The images from the eight adult human volunteers are analyzed using LUM, APM+, APM* (first step of intravascular fluence correction only), and APM, respectively, and the estimates are plotted as in Figure 3e. The resulting **a,** estimated radial venous sO_2_ values (sample size: 30) and **b,** differences between the accompanying radial vein estimates (sample size: 15) from each method are plotted. Each step of intravascular fluence correction progressively improves the estimation performance. |
| --- |


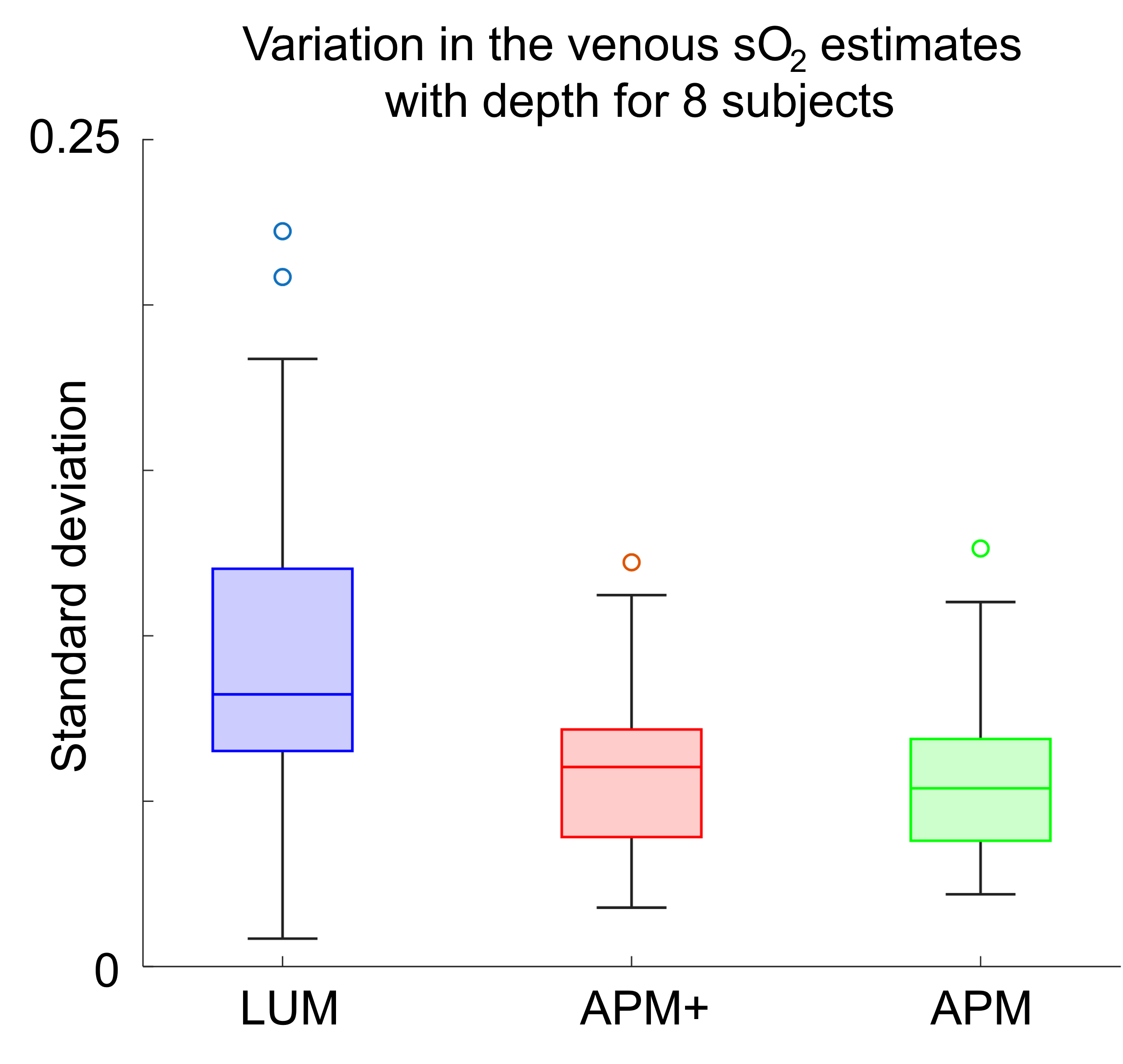


| **Figure S2\| Comparison of in-vivo consistency of LUM, APM+, and APM.**  Plot of the standard deviation with depth (see Figure 4d) of the LUM, APM+, and APM venous sO_2_ estimates for 8 healthy adult human subjects (15 wrist images, 30 vessels). Outliers are samples that are more than 1.5 times the interquartile range away from the first or third quartiles. The variations of the APM and APM+ estimates are noticeably lower than the LUM ones. However, the variations of APM and APM+ are not significantly different from each other, as confirmed by a paired-samples t-test (p > 0.05). This is not surprising since both methods rely on arterial calibration, which makes their errors robust to the overlying tissue thickness. |
| --- |

**Note S1| Impact of skin tone on APM+.**

Since APM+ calibrates the fluence ratio using an artery inside the tissue, one of its key advantages is that it is free of skin-tone bias. To demonstrate this, we conducted numerical simulations using a 2D digital phantom modeled on the wrist. The structure of the digital phantom is shown in Figure S3. The phantom consists of a 0.2 mm thin epidermal layer containing melanin, a few randomly placed small vessels below the skin, and a vein-artery-vein structure (modeled on the radial artery and its venae comitantes) at a depth of ∼5 mm from the skin, each with a vessel diameter of 1 mm. The lateral (center-to-center) distance between the artery and each vein was fixed at 1.25 mm, and the depth of each vein relative to the artery was randomly chosen within ±0.5 mm. The background tissue was assumed to have a blood volume fraction of 5%, an sO_2_ of 65%, and a water volume fraction of 10%, respectively. The sO_2_s of the artery and veins were set to 97% and 70%, respectively. Based on literature values^13^, four sets of melanin absorption parameters were chosen, corresponding to no skin absorption and Fitzpatrick skin types I–II, III–IV, and V–VI, respectively. The parameters corresponding to these four cases, along with the LUM and APM+ estimates of venous sO_2,_ are shown in Figure S3. We see here that while the LUM estimates, apart from being highly erroneous (>20%), exhibit considerable variation with the skin type, the APM+ estimates are remarkably consistent (variation of the order of 10^-3^%). We verified this consistency across ten realizations of the digital wrist phantom.


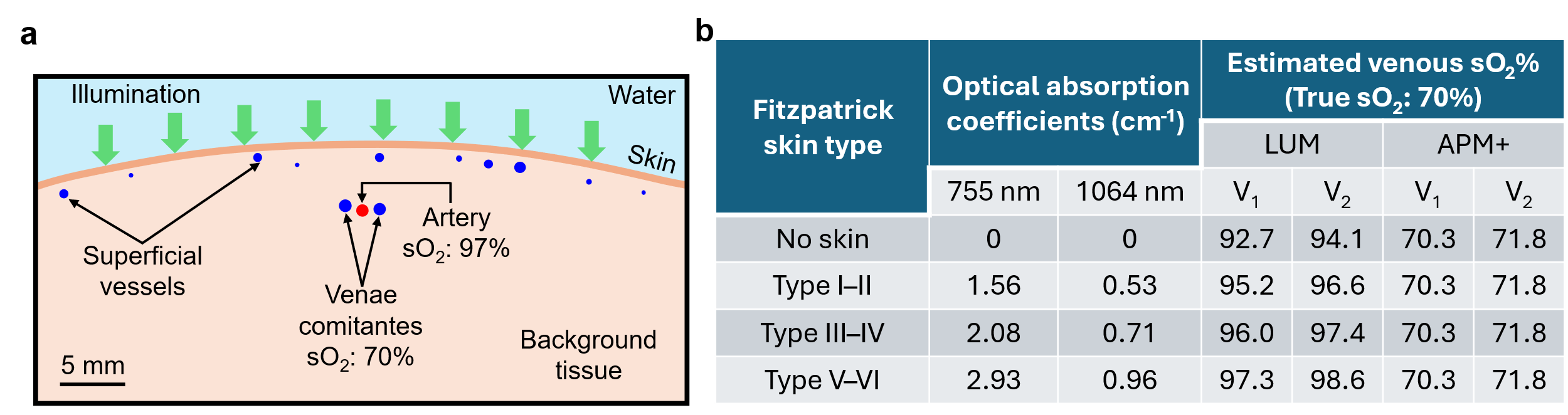


**Figure S3| Demonstration of the skin tone bias-free nature of APM+. a,** Digital wrist phantom structure. **b,** Optical absorption coefficients for the four skin types considered, along with the LUM and APM+ estimates of venous sO_2_ for each case. The LUM estimates, apart from being highly erroneous (>20%), exhibit considerable variation with skin type, whereas the APM+ estimates are highly consistent.

**Note S2| Error analysis of APM.**

There are two primary sources of errors in APM: erroneous arterial oxygenation (SaO_2_) and fluence ratio heterogeneity. To understand the effect of these two sources of error on APM, we consider the first-order Taylor expansion of Eq. (8) in the manuscript. For simplicity in notation, let $f\left( x \right)={\text{s}\hat{\text{O}}}_{2}\left( \vec{r} \right)$, where $x=\hat{\mu}_{R}\left( \vec{r} \right)$ (defined in Eq. (7)).

|  | $f\left( x \right)=\left( 1-\frac{\varepsilon_{\text{HbO}_{2}}\left( \lambda_{2} \right)x-\varepsilon_{\text{HbO}_{2}}\left( \lambda_{1} \right)}{\varepsilon_{\text{Hb}}\left( \lambda_{2} \right)x-\varepsilon_{\text{Hb}}\left( \lambda_{1} \right)} \right)^{-1}=\frac{ax+b}{cx+d}\text{,}$ | (S1) |
| --- | --- | --- |

where $a=\varepsilon_{\text{Hb}}\left( \lambda_{2} \right)$, $b=-\varepsilon_{\text{Hb}}\left( \lambda_{1} \right)$, $c=\varepsilon_{\text{Hb}}\left( \lambda_{2} \right)-\varepsilon_{\text{HbO}_{2}}\left( \lambda_{2} \right)$, and $d=-(\varepsilon_{\text{Hb}}\left( \lambda_{1} \right)-\varepsilon_{\text{HbO}_{2}}\left( \lambda_{1} \right))$. Let the true value of $x$ be $x_{0}=\mu_{R}$. The first-order Taylor expansion of $f(x)$ around $x_{0}$ is

|  | $f\left( x \right)=f\left( x_{0} \right)+f^{'}\left( x_{0} \right)\left( x-x_{0} \right)\mathcal{+O}\left( \left( x-x_{0} \right)^{2} \right).$ | (S2) |
| --- | --- | --- |

The higher-order terms in Eq. (S2) can be ignored when $\left| x-x_{0} \right|\ll\left| \frac{2f^{'}\left( x_{0} \right)}{f^{''}\left( x_{0} \right)} \right|$. The first and second derivatives of $f(x)$ are $f^{'}\left( x \right)=\frac{ad-bc}{\left( cx+d \right)^{2}}$ and $f^{''}\left( x \right)=-\frac{2c(ad-bc)}{\left( cx+d \right)^{3}}$. Plugging these into Eq. (S2), we get

|  | $f\left( x \right)\approx f\left( x_{0} \right)+\frac{ad-bc}{\left( cx_{0}+d \right)^{2}}\left( x-x_{0} \right)\text{,} \left\vert x-x_{0} \right\vert\ll\left\vert x_{0}+\frac{d}{c} \right\vert.$ | (S3) |
| --- | --- | --- |

Since $p_{0}\left( \vec{r},\lambda\right)\propto F\left( \vec{r},\lambda\right)\mu_{a}\left( \vec{r},\lambda\right)$, Eq. (7) can be rewritten as

|  | $x=\left( \frac{F_{R}\left( \vec{r} \right)}{F_{R}\left( \vec{r}_{\text{art}} \right)} \right)\left( \frac{\tilde{\mu}_{R}\left( \vec{r}_{\text{art}} \right)}{\mu_{R}\left( \vec{r}_{\text{art}} \right)} \right)x_{0}\text{,}$ | (S4) |
| --- | --- | --- |

where $F_{R}\left( \vec{r} \right)=\frac{F\left( \vec{r},\lambda_{1} \right)}{F\left( \vec{r},\lambda_{2} \right)}$ and $\tilde{\mu}_{R}\left( \vec{r}_{\text{art}} \right)$ is the arterial absorption coefficient ratio obtained using the assumed/measured SaO_2_. Eq. (S4) captures the effects of fluence ratio heterogeneity and SaO_2_ error, respectively, on APM.

For $\lambda_{1}=$ 755 nm, $\lambda_{2}=$1064 nm, and a venous sO_2_ of 70%, we get

|  | $f\left( x \right)\approx0.7-0.419\left( \frac{F_{R}\left( \vec{r} \right)}{F_{R}\left( \vec{r}_{\text{art}} \right)}\cdot\frac{\tilde{\mu}_{R}\left( \vec{r}_{\text{art}} \right)}{\mu_{R}\left( \vec{r}_{\text{art}} \right)} -1 \right)\text{,} 1.422\leq x\leq2.113.$ | (S5) |
| --- | --- | --- |

The domain $1.422\leq x\leq2.113$ indicates that the linear approximation of $f\left( x \right)$ is valid within an sO_2_ estimation error of approximately $\pm$10%. We can consider the effect of the two sources of errors on APM independently, since they appear in a separable way in Eq. (S4).

1. Fluence ratio heterogeneity: Assuming that there is no error in the SaO_2_, if we define the region of APM’s spatial validity as the locations where the sO_2_ estimation error is less than 5%, this places a limit on the fluence ratio heterogeneity of $\sim$12% (relative to $F_{R}\left( \vec{r}_{\text{art}} \right)$). If the maximum acceptable sO_2_ estimation error is 10%, the fluence ratio heterogeneity needs to be roughly within 24%. To visualize this region of validity, we conducted some numerical simulations using a 2D digital wrist phantom, comprising an elliptical tissue boundary (similar to the structure in Figure S3a). The background tissue was assumed to have a blood volume fraction of 5%, an sO_2_ of 65%, and a water volume fraction of 10%, respectively, based on the literature^13^. We simulated three different scenarios. First, we considered homogeneous tissue without any blood vessels. In the second scenario, we incorporated several vascular inclusions to simulate the shadowing induced by superficial blood vessels in the wrist. The vessel positions and radii were chosen randomly. In the third case, we included two large elliptical heterogeneities to simulate lateral heterogeneities in tissue. The optical absorption and scattering coefficients of the respective heterogeneous regions were obtained by multiplying the corresponding coefficients of the background tissue by a uniform random variable between 0.5 and 1.5.

For each case, we obtain the fluence maps corresponding to 755 nm and 1064 nm illuminations, respectively, and compute the ratio of the two. We pick a point within the medium as the calibration point and assume that its fluence ratio is known (analogous to an artery in APM). Then, assuming a true venous sO_2_ of 70%, we can convert the fluence ratio heterogeneity into an sO_2_ estimation error using Eq. (S5). The sO_2_ estimation maps for the three cases are shown in Figure S4. For each case, the absolute sO_2_ estimation error map was computed and thresholded at 10%, beyond which Eq. (S5) does not hold.

As expected, for the homogeneous case, the fluence ratio is quite uniform laterally. Along depth, the sO_2_ estimation error can be maintained within 5% and 10% for a depth range of 3.1 mm and 6 mm, respectively. For the second case, with vascular inclusions, we expect that the shadowing from these vessels will affect the fluence ratio. However, from the center panel of Figure S4, we see that this effect, while noticeable, is also small (<5% around each vessel) and local. This illustrates that while superficial vessels will affect the sO_2_ estimation accuracy using APM, the error induced is small and contained mostly within a very small region around the vessel. Moreover, the lateral extent of the validity of the method is not significantly affected by the vascular inclusions. However, along depth, the slice thicknesses at 5% and 10% errors are reduced from the first case to 2.4 mm and 5.1 mm, respectively. Finally, for the large inhomogeneities, while we clearly see the effect of the two inhomogeneities (shown by the dashed green ellipses) on the estimation error, the reduction in the estimation accuracy along both the lateral and depth direction is not significant despite the large variation between the optical properties of the background tissue and the inclusions. The slice thicknesses along depth at 5% and 10% estimation error for the third case are the highest amongst the three at 3.5 mm and 6.6 mm, respectively. Laterally, the width at 5% estimation error is over 1 cm for all three cases. Lastly, we also experimentally verified that the lateral spatial range over which the fluence ratio (for 755 nm and 1064 nm illumination) varies by less than 12% (and the sO_2_ error is less than 5%) is well over 1 cm for both chicken and porcine tissue samples of thicknesses up to 15 mm.

1. SaO_2_ error: Assuming that the arterial fluence ratio calibration is perfect, i.e., $\frac{F_{R}\left( \vec{r} \right)}{F_{R}\left( \vec{r}_{\text{art}} \right)}=1$, for a true SaO_2_ of 97%, true venous sO_2_ of 70%, and assumed/measured SaO_2_s between 95% and 100%, $x$ varies between 1.63 and 1.86. Therefore, the first-order approximation is valid here. Correspondingly, from Eq. (S5), the estimated venous sO_2_ changes from 67.8% (for an SaO_2_ = 95%) to 73.2% (SaO_2_ = 100%) in an approximately linear fashion with respect to the assumed SaO_2_.


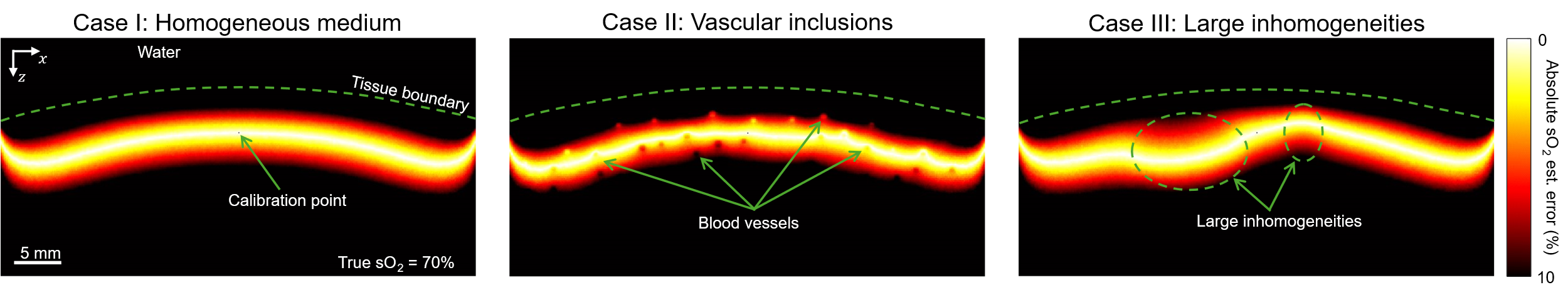


**Figure S4| Analysis of the sO_2_ estimation error due to fluence ratio variation.** Three cases are simulated. First, a homogeneous tissue is considered. The tissue boundary is indicated using a green dashed line. Here, the fluence ratio is quite uniform laterally. Along depth, the sO_2_ estimation error can be maintained within 5% and 10% for a depth range of 3.1 mm and 6 mm, respectively. In the second case, we consider numerous vascular inclusions in the tissue to simulate shadowing from a superficial vessel. In this case, the error induced due to the vessels is small and contained mostly within a small region around the vessel. Moreover, the lateral extent of the validity of the method is not significantly affected by the vascular inclusions. However, along depth, the slice thicknesses at 5% and 10% errors are reduced slightly from the first case to 2.4 mm and 5.1 mm, respectively. In the third case, we include two large inhomogeneities. Here, while we clearly see the effect of the two inhomogeneities (shown by the dashed green ellipses) on the estimation error, the reduction in the estimation accuracy along both the lateral and depth direction is not significant despite the large variation between the optical properties of the background tissue and the inclusions. The slice thicknesses along depth at 5% and 10% estimation error for the third case are the highest amongst the three at 3.5 mm and 6.6 mm, respectively. Laterally, the width at 5% estimation error is over 1 cm for all three cases.

**Note S3| Choosing the best wavelengths for APM.**

To conduct an analysis of the impact of the choice of wavelengths on APM’s accuracy, we rely on the Taylor expansion in Eq. (S3). Combining this with Eq. (S4), we have

|  | $f\left( x \right)\approx f\left( x_{0} \right)+\frac{ad-bc}{\left( cx_{0}+d \right)^{2}}\left( \frac{F_{R}\left( \vec{r} \right)}{F_{R}\left( \vec{r}_{\text{art}} \right)}\cdot\frac{\tilde{\mu}_{R}(\vec{r}_{\text{art}})}{\mu_{R}(\vec{r}_{\text{art}})}-1 \right)x_{0}\text{,} \left\vert x-x_{0} \right\vert\ll\left\vert x_{0}+\frac{d}{c} \right\vert.$ | (S6) |
| --- | --- | --- |

The second term of Eq. (S6) can be analyzed for different wavelength pairs and for each error source independently, as shown below.

1. Fluence ratio heterogeneity: First, we assume that the SaO_2_ is known exactly, i.e., $\tilde{\mu}_{R}(\vec{r}_{\text{art}})=\mu_{R}(\vec{r}_{\text{art}})$. Then, the quantity $\beta\left( \lambda_{1},\lambda_{2} \right)=\frac{ad-bc}{\left( cx_{0}+d \right)^{2}}x_{0}$ represents the sensitivity of a given wavelength pair to fluence ratio heterogeneity. Figure S5a shows a plot of this sensitivity for wavelengths between 700 and 1100 nm. If we have no further knowledge regarding the tissue composition, we may have to rely on this sensitivity to pick the best wavelength pair. However, assuming a homogeneous background tissue, we can rely on diffusion theory to gain further insight. Assuming a one-dimensional scenario with an infinitely broad time-independent light source, $\delta(z)$, the fluence in an infinite homogeneous medium varies with depth as $exp(-\mu_{\text{eff}}(\lambda)z)$, where $\mu_{\text{eff}}(\lambda)$ is the effective optical attenuation coefficient. For arterial calibration at a depth of $z_{0}$, we have $\frac{F_{R}\left( z_{0}+\Delta z \right)}{F_{R}(z_{0})}=exp(\left( \mu_{\text{eff}}\left( \lambda_{2} \right)-\mu_{\text{eff}}\left( \lambda_{1} \right) \right)\Delta z)$. Plugging this into Eq. (S6), we get the following relation between the estimated sO_2_ error $\Delta f$ and depth $\Delta z$.

|  | $\Delta f=\frac{ad-bc}{\left( cx_{0}+d \right)^{2}}\left( \exp\left( \left( \mu_{\text{eff}}(\lambda_{2})-\mu_{\text{eff}}(\lambda_{1}) \right)\Delta z \right)-1 \right)x_{0}.$ | (S7) |
| --- | --- | --- |

For a desired maximum estimation error, $\Delta f$, the depth validity can be computed using Eq. (S7) for different wavelength pairs, and the performance for different pairs can be estimated. Assuming a blood volume fraction of 5%, an sO_2_ of 65%, a water volume fraction of 10%, and an anisotropy factor of 0.9 for background tissue, we compute the distance from the calibration location, $\left| \Delta z \right|$, at which $\Delta f=$ 5% is attained and plot it in Figure S5b. Curiously, we see here that there are certain wavelength combinations for which $\left| \Delta z \right|>$ 1 cm. It turns out that these are wavelengths at which the effective attenuation coefficients are very similar, as shown in Figure S5c. However, since the wavelengths at which this equality occurs change significantly with the background tissue sO_2_, it may be challenging in practice to pick the optimal wavelengths even for a homogeneous tissue structure. Note that for some wavelength pairs in Figure S5a–c, especially those that are very close, the linear approximation in Eq. (S6) may not be valid.

1. SaO_2_ error: To study this, we assume that $\frac{F_{R}\left( \vec{r} \right)}{F_{R}\left( \vec{r}_{\text{art}} \right)}=1$. Therefore, we have

|  | $\Delta f=\frac{ad-bc}{\left( cx_{0}+d \right)^{2}}\left( \frac{\tilde{\mu}_{R}(\vec{r}_{\text{art}})}{\mu_{R}(\vec{r}_{\text{art}})}-1 \right)x_{0}.$ | (S8) |
| --- | --- | --- |

Once again, we compute the absolute sO_2_ estimation error for a $\pm$2% error in the assumed SaO_2_ (true SaO_2_ = 97%) for different wavelength pairs between 700 and 1100 nm and show it in Figure S5d. The impact of the wavelength choice on APM error due to SaO_2_ error is much weaker than that of the fluence ratio heterogeneity.


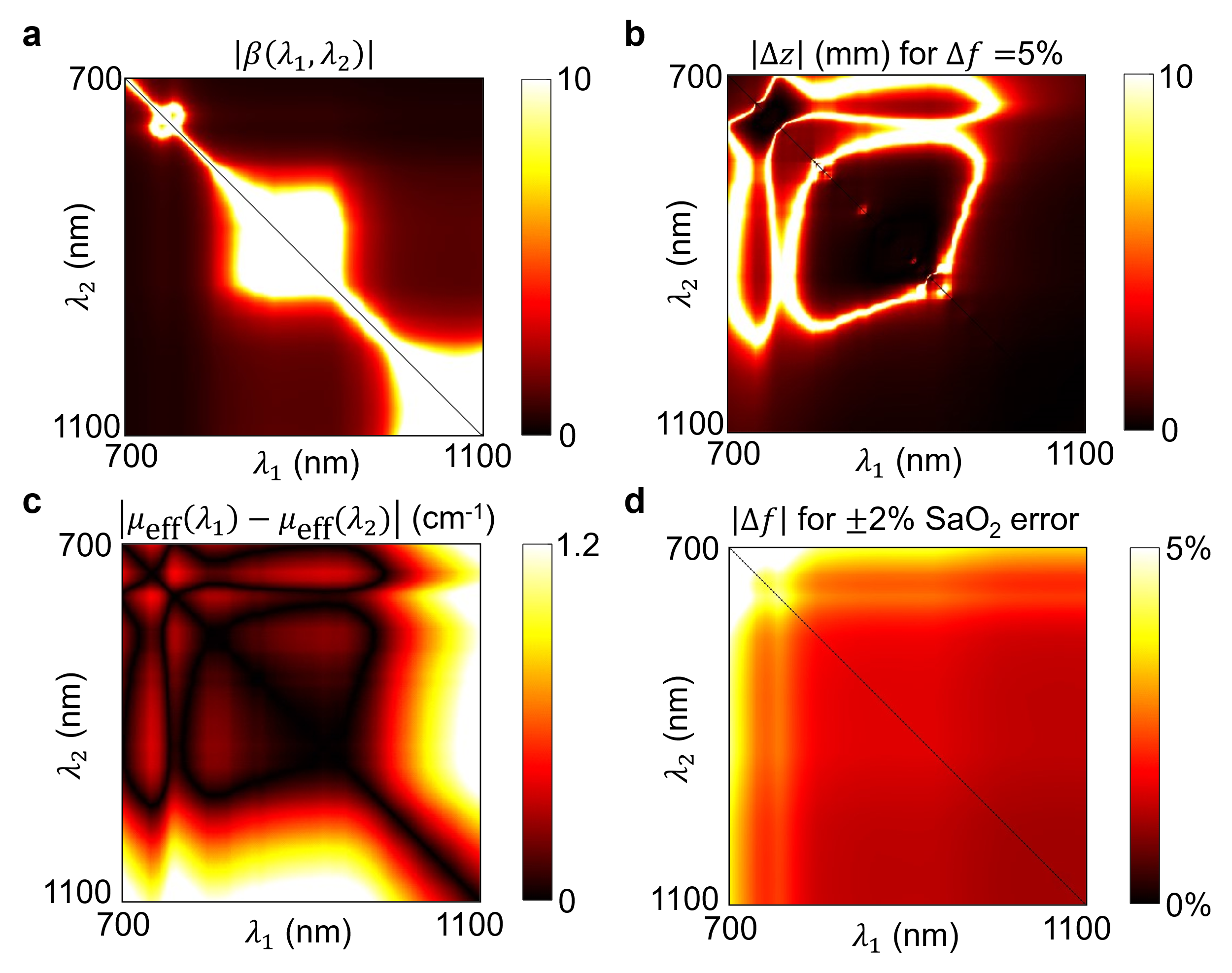


**Figure S5| Impact of wavelength choice on APM. a,** Plot of APM’s error sensitivity to fluence ratio heterogeneity, $\beta(\lambda_{1},\lambda_{2})$, for different wavelength pairs between 700 and 1100 nm. **b,** Plot of the distance from the calibration location, $\left| \Delta z \right|$, at which $\Delta f=$ 5% is attained, for a homogeneous background tissue (blood volume fraction = 5%, sO_2_ = 65%, water volume fraction = 10%, anisotropy factor = 0.9). Note that for some wavelength pairs, especially those that are very close, the linear approximation in Eq. (S6) may not be valid. **c,** Plot of the absolute difference between the effective attenuation coefficients for different wavelength pairs. The wavelengths for which this difference is very low correspond to pairs for which $\left| \Delta z \right|>$ 1 cm in **b**. **d,** Plot of the absolute sO_2_ estimation error for a $\pm$2% error in the assumed SaO_2_ (true SaO_2_ = 97%) for different wavelength pairs. The colorbars of the plots are saturated at their respective indicated maximums to accommodate the high dynamic range and highlight variations in the meaningful lower-value regions.

**Note S4| Analysis of the in-vivo accuracies of APM+ and LUM.**

In the absence of an independent ground truth for venous sO_2_ in our in-vivo experiments, only the precision or consistency of the method can be computed in-vivo. However, under some assumptions, we can compare the accuracies of the two methods (LUM and APM+). Let the true and estimated sO_2_ using any given method be the random variables $f$ and $\hat{f}$, respectively, related as $\hat{f}=f+e$, where $e$ is the estimation error. The bias and variance of the error are $\bar{e}=E[e]$ and $\text{Var}\left( e \right)=E\left[ e^{2} \right]-\bar{e}^{2}$, respectively. Since $E[e^{2}]$ is the mean squared error (MSE), we reach the familiar relationship, $\text{MSE}=\text{Var}\left( e \right)+\bar{e}^{2}$.

For the estimation results presented in Figure 3e, we have ${\bar{\hat{f}}}_{\text{LUM}}\approx0.691$, $\text{Var}\left( \hat{f}_{\text{LUM}} \right)\approx0.0433$, ${\bar{\hat{f}}}_{\text{APM+}}\approx0.712$, and $\text{Var}\left( \hat{f}_{\text{APM+}} \right)\approx0.0108$ ($\approx$ because these are the sampled estimates of the respective statistics). Since ${\bar{\hat{f}}}_{\text{LUM}}\approx{\bar{\hat{f}}}_{\text{APM+}}$ and $\bar{e}=\bar{\hat{f}}-\bar{f}$, we have $\bar{e}_{\text{LUM}}\approx\bar{e}_{\text{APM+}}$, i.e., the error biases of LUM and APM+ are approximately equal. Thus, we have $\text{MSE}_{\text{LUM}}-\text{MSE}_{\text{APM+}}\approx\text{Var}\left( e_{\text{LUM}} \right)-\text{Var}\left( e_{\text{APM+}} \right)$.

Assuming that the ground truth, $f$, and the estimation errors, $e_{\text{LUM}}$ and $e_{\text{APM+}}$, are independent, $\text{Var}\left( \hat{f}_{\text{LUM}} \right)=\text{Var}\left( f \right)+ \text{Var}\left( e_{\text{LUM}} \right)$ and $\text{Var}\left( \hat{f}_{\text{APM+}} \right)=\text{Var}\left( f \right)+ \text{Var}\left( e_{\text{APM+}} \right)$. Subtracting the two, we get $\text{Var}\left( e_{\text{LUM}} \right)-\text{Var}\left( e_{\text{APM+}} \right)=\text{Var}\left( \hat{f}_{\text{LUM}} \right)- \text{Var}\left( \hat{f}_{\text{APM+}} \right)=0.0325$. Finally, under the assumption of approximately equal biases of LUM and APM+, we get $\text{MSE}_{\text{LUM}}-\text{MSE}_{\text{APM+}}\approx0.0325$, which shows that APM+ is indeed more accurate than LUM.

Even if we do not assume that the biases are approximately equal, for all $\bar{f}$ between [0,1], we still have $\text{MSE}_{\text{LUM}}>\text{MSE}_{\text{APM+}}$. Therefore, the only assumption for the above proof to hold is regarding the independence of the ground truth and the error, $f$ and $e$, respectively. Although this is not strictly true due to the boundedness of the ground truth (between 0 and 1), we can verify from the phantom results in Figure 2c, where we vary the ground truth between 0% and 100%, that the two methods do not exhibit the severe, opposing correlations with the ground truth required to reverse the above conclusion to $\text{MSE}_{\text{LUM}}<\text{MSE}_{\text{APM+}}$. In fact, we find that the LUM errors exhibit a negative correlation (Pearson correlation coefficient/PCC$\approx-$0.75) with the ground truth, whereas the APM+ errors are very weakly correlated (PCC$\approx$0.02), which indicates that the independence assumption yields a conservative lower bound, and the actual difference between the MSEs is even larger.
